# Improved HHT-microstate analysis of EEG in nicotine addicts

**DOI:** 10.3389/fnins.2023.1174399

**Published:** 2023-05-24

**Authors:** Xin Xiong, Jiannan Feng, Yaru Zhang, Di Wu, Sanli Yi, Chunwu Wang, Ruixiang Liu, Jianfeng He

**Affiliations:** ^1^Faculty of Information Engineering and Automation, Kunming University of Science and Technology, Kunming, China; ^2^College of Physics and Electronic Engineering, Hanshan Normal University, Chaozhou, China; ^3^Department of Clinical Psychology, Second People's Hospital of Yunnan, Kunming, China

**Keywords:** addictions, improved HHT-microstate, EEG, frequency band, detect

## Abstract

**Background:**

Substance addiction is a chronic disease which causes great harm to modern society and individuals. At present, many studies have applied EEG analysis methods to the substance addiction detection and treatment. As a tool to describe the spatio-temporal dynamic characteristics of large-scale electrophysiological data, EEG microstate analysis has been widely used, which is an effective method to study the relationship between EEG electrodynamics and cognition or disease.

**Methods:**

To study the difference of EEG microstate parameters of nicotine addicts at each frequency band, we combine an improved Hilbert Huang Transformation (HHT) decomposition with microstate analysis, which is applied to the EEG of nicotine addicts.

**Results:**

After using improved HHT-Microstate method, we notice that there is significant difference in EEG microstates of nicotine addicts between viewing smoke pictures group (smoke) and viewing neutral pictures group (neutral). Firstly, there is a significant difference in EEG microstates at full-frequency band between smoke and neutral group. Compared with the FIR-Microstate method, the similarity index of microstate topographic maps at alpha and beta bands had significant differences between smoke and neutral group. Secondly, we find significant class × group interactions for microstate parameters at delta, alpha and beta bands. Finally, the microstate parameters at delta, alpha and beta bands obtained by the improved HHT-microstate analysis method are selected as features for classification and detection under the Gaussian kernel support vector machine. The highest accuracy is 92% sensitivity is 94% and specificity is 91%, which can more effectively detect and identify addiction diseases than FIR-Microstate and FIR-Riemann methods.

**Conclusion:**

Thus, the improved HHT-Microstate analysis method can effectively identify substance addiction diseases and provide new ideas and insights for the brain research of nicotine addiction.

## 1. Introduction

Substance addiction is a chronic relapsing disease, which refers to the adaptation and dependence of individuals after long-term abuse of harmful substances (World Health Organization, 2009). It includes drug addiction and other mental addictions such as alcohol, nicotine, and caffeine. The root cause is the long-term adaptation of the brain to addictive substances, which makes it difficult for individuals to give up due to the escalating behavior of substance intake, even though they have been aware of the negative effects (Pengfei et al., 2019). Substance addiction can lead to a range of diseases, such as lung cancer, iron-deficiency heart disease and esophageal cancer, and cause an enormous emotional, financial and medical burdens on individuals and society. Previous studies on the cognitive function of addicts with different substances have shown that addiction have an impact on cognition. For example, addicts have impaired executive control function, increased impulsivity (Fulton and Charlotte, 2009), decreased decision-making ability (Zernig et al., 2007), and strong memories related to addiction cues (Robbins et al., 2008; Yan Xue et al., 2017); Meanwhile, drug addicts have abnormal sleep structure (Conroy and Arnedt, 2014), whose sleep stages affected by various addictive substances (Colrain et al., 2014). In addition, addiction theory has also shown that a common character of substance addiction is drug cue response, which means that compared with non-addicts, addicts show significantly different physiological and psychological responses when they face with smoke cues or neutral cues (Linyuan and Xiaoyi, 2005). Moreover, the responsiveness of addicts to cigarette-related cues is also the main factor of relapse (Wei et al., 2017), which means that the brain response of addicts to cigarette cues may predict their ability to give up smoking continually. At present, the main treatment methods of substance addiction are drug therapy, psychotherapy, physical therapy, and neutral feedback therapy. In recent years, the technique of Brain Computer Interface (BCI) from the perspective of electrophysiology has been proposed and applied to the research of addiction. BCI is a communication system built between the brain and other external devices, rather than relying on the brain transmission pathway composed of peripheral nerves and muscles, which is a new way of human-computer interaction. By using a non-invasive, cheap and powerful tool, Electroencephalogram (EEG), it can record the configuration of brain electric fields produced by the coordination of different nerve combinations and have a high temporal resolution (Arshad et al., 2022; Totev et al., 2023). Besides, with the development of the computer technology, some relevant and novel algorithms including Generative Adversarial Network (GAN), Convolutional Neural Networks (CNN) were employed for features extraction at EEG bands or to explore the potential neutral mechanism of the brain (Hu et al., 2020; Prasanth et al., 2020; Yan et al., 2020), and also made great contributions in some specific states or diseases such as Parkinson, depression and epilepsy (Uyulan et al., 2020; Chu et al., 2021; Gabeff et al., 2021). On this basis, studies showed that the brain of substance addict has abnormal functions and structural changes (Bjork and Gilman, 2014). Therefore, many researchers have processed the cognitive function of the brain in different states and collected signals from the cerebral cortex to analyze the mechanism of addiction.

Previous investigations and studies on EEG signals of addicts have shown that there are some qualitative and quantitative changes in EEG signals of these addicts, including EEG coherence, frequency domain features and nonlinear features, and EEG source localization. As a measurement of brain network, coherence reflects functional connectivity and activity synchronization among brain regions (Franken et al., 2004) and has advantages in terms of high temporal resolution and measurement of brain networks among neuron populations. Compared with non-addicted people, EEG coherence in addicted people is significantly enhanced (Yan Xue et al., 2017) and significantly correlate with the changes in smoking cravings (Littel et al., 2009), which is advisable to explore the changes in brain activity related to addiction. In the frequency domain, there are less alpha EEG rhythm and more beta EEG rhythm in the addicted people, and many delta-theta rhythms with low amplitude in the central brain region (Benos and Kapinas, 1980; Olivennes et al., 1983; Gekht et al., 2002); addicts have higher EEG correlation dimension than non-addicts, which can reflect the attention deficit of addicted people (Světlák et al., 2010). Furthermore, the analysis method of EEG source localization has also been applied to research the mechanism of addiction and solved the problem of observing the difference of brain activity and locating deep source error in high temporal and spatial resolution (Pascualmarqui et al., 1994). In addition, the rank-based feature selection method was used to assign weight values to EEG features such as the interhemispheric coherence and spectral power at EEG bands of patients with alcohol disorders, which obtain the better accuracy with the classification of the most discriminative features (Mumtaz et al., 2017). Meanwhile, study also found that the theta band (4–8 Hz) between the frontal and posterior cortical regions had a high level of synchronization in the brain of drug addicts according to the connectivity of subband cortical network which was calculated by synchronization likelihood algorithm (Coullaut-Valera et al., 2014).

EEG microstate is one of the methods to determine and quantify the oscillatory activity and dynamic characteristics of the cerebral cortex. It was first proposed by Lehmann, who regarded the multi-channel spontaneous EEG signals as a series of EEG topographic maps changing over time. Each EEG topographic map is the superposition of the effects of all the sources that are instantly active at present and is a global measure of instantaneous EEG activity (Lehmann et al., 1987; Lehmann, 1994). It reveals that the distribution of brain electrical activity does not change continuously but discretely over time. The topological structure of one EEG topographic map does not smoothly change to another structure, but stays in a quasi-stable state for about 80–120 ms, and then suddenly changes to another structure. Several EEG topographic maps with the same topological structure are classified as a class of microstate (Arjun et al., 2014). In the literature of microstate analysis, generally four different microstate, typically labeled from A to D, respectively correspond to the activities of auditory network, visual network, prominence network and attention network (Britz et al., 2010) and can usually explain more than 80% of the variance present in the EEG data (Lehmann et al., 2005; Seitzman et al., 2017). The temporal parameters of microstate include global interpretation variance, mean duration, occurrence and coverage of microstates, which provide new avenues for quantifying cortical oscillatory activity with functional relevance. The changes of these parameters can reflect the impact of diseases on the brain, such as Parkinson’s disease (John et al., 2023), dementia (Grieder et al., 2016), schizophrenia (Andreou et al., 2014) and Alzheimer’s disease (Strik et al., 1997). Besides, there are also studies on the identification of epilepsy (Kiran et al., 2018) and motor imagination (Weifeng et al., 2017) by using microstate parameters, and the accuracy is relatively high. However, most previous studies on EEG of addicted subjects were based on the analysis of brain network and EEG characteristics. Therefore, using microstate analysis to compare different EEG of addicts is a valuable method to analysis and detect substance addiction.

Previous studies have shown that the dynamics of microstates in the time domain are correlated with those in the spectrum domain. Milz et al. (2017) found that there was a consistent relationship between intra-band microstates and power, which meant that the intra-cortical intensity and spatial distribution of alpha frequency band were determined. Traditional microstate analysis method lacks frequency domain information (Koenig et al., 2018), which affects the conclusion of correlation between microstate and spectrum domain. In order to solve this problem, Ehtasham et al. (2019) used the empirical mode decomposition (EMD) and instantaneous frequency model in the Hilbert Huang Transform (HHT) method to extract the spectral features of microstates in time series. This method can preserve the local spectral properties of the original data in time domain, and does not require prior characteristic information of data, or as in the case of other decomposition methods such as Fourier, or wavelet analysis, it does not assume a pre-determined set of basis functions (Daubechies et al., 2011; Thakur et al., 2013). Therefore, in this paper, an improved HHT-Microstate method was used to research the EEG of nicotine addicts. By preserving the instantaneous properties of the data in the spectrum domain, the microstate time series was analyzed to evaluate the instantaneous changes of the spectral features of the EEG data.

In this study, we selected two different types of nicotine addiction EEG data and used the improved HHT method to divide the EEG data into five frequency bands, including delta band (0.5–4 Hz), theta band (4–8 Hz), alpha band (8–12 Hz), beta band (12–30 Hz), and gamma band (30–40 Hz). By comparing the differences of the microstate parameters between the two types of tasks at each frequency band, we selected the microstate parameters with significant differences as features to detect different types of nicotine addiction. At the same time, in order to highlight the superiority of the improved HHT-Microstate method, the frequency band division method and EEG feature analysis method are compared with Finite Impulse Response (FIR) method and EEG Riemann distance method, which includes FIR-Microstate method, HHT-Riemann method and FIR-Riemann method. Finally, we proved that the improved HHT-Microstate method is superior to other methods and can detect and identify different addiction states more effectively.

## 2. Materials and methods

### 2.1. Method

Our experiments in this paper are carried out on the platform of Matlab_2019 and the corresponding toolbox of EEGLAB_2019. The main method has two parts. Firstly, the data is time-frequency decomposition by using the Empirical Mode Decomposition (EMD) and instantaneous frequency model. Secondly, microstate analysis method is applied to each frequency band for the extraction of microstate topographic maps and microstate parameters at each frequency band.

#### 2.1.1. Time-frequency analysis—improved Hilbert Huang transform

The obstacle to finding a correlation between microstates and spectrum is to correlate microstates in different temporal resolutions with spectral analysis. On the one hand, the EEG microstate analysis is carried out in the time domain and determine the EEG data and similarity index of the given segmentation for each instance; On the other hand, traditional spectrum analysis methods require at least a period to calculate the spectral power of any given frequency band. Therefore, in order to solve this obstacle, the EMD and instantaneous frequency models in HHT (Huang, 1998) were used for time-frequency analysis (Huang et al., 2009; Liu et al., 2022). However, in the process of decomposition, the traditional EMD will cause problems of mode aliasing, which makes the component lose the single feature scale feature. Ensemble Empirical Mode Decomposition (EEMD) model has been optimized for EMD, but the added Gaussian white noise will remain and affect the result. To solve the problem of mode aliasing in EMD and reconstruction error or low computational efficiency in EEMD, a CEEMDAN method is used in this paper. By adding adaptive white noise to the EEMD, errors in signal reconstruction can be reduced and high computational efficiency can be ensured. Besides, it could maintain the original temporal resolution while transforming time-domain data into time-frequency domain data. The main steps of CEEMDAN are as follows:

(1) Signal can obtain several Intrinsic Mode Functions (IMFs) after EMD, and each IMF must satisfy two restrictions: ① The difference between the number of extreme points and zero points is not more than 1; ② The mean value between the local maximum envelope and the local minimum envelope at any time is 0.

Add white noise βAj(t) to the original signal x(t), where β is the standard noise and j is the number of noises added. The newly constructed signal is Z(t) = x(t) + βAj(t). The first order component of CEEMDAN is:


(1) $$IMF_{1}=\frac{1}{n}\overset{n}{\underset{j=1}{\sum }}IMF_{1}^{j}$$

The remainder is r1 = Z(t) − IMF_1_.

(2) The original signal of the second IMF component is Z(t) = r1 + βAj(t), after decomposition, it can obtain:

(2) $$IMF_{2}=\frac{1}{2n}\overset{2n}{\underset{j=1}{\sum }}IMF_{2}^{j}$$

The remainder is r2 = Z(t) − IMF_2_.

(3) Next, repeat step (1) and (2) until the decomposition is complete. m IMF components are obtained, and the residual is:

(3) $$r=x\left(t\right)-\overset{m}{\underset{i=1}{\sum }}IMF_{i}$$

(4) The reconstruction formula is:

(4) $$Z\left(t\right)=r+\overset{m}{\underset{i=1}{\sum }}IMF_{i}$$

Due to the existence of false IMF components in the process of EMD decomposition, these false IMF components should be eliminated in practical application. The correlation between the real IMF component and the original signal is greater than those in false component, and the proportion of the real IMF component is larger than those in false IMF component. At present, the commonly used methods to eliminate false IMF components include correlation coefficient method, gray correlation degree method, mutual Information method, energy ratio method and K-S test method. Gray correlation degree method and K-S test method can better distinguish the false IMF component for single component signals, but it is difficult to distinguish the complex signals with multiple components (Yang et al., 2013). Correlation coefficient method and energy ratio method have great amplitude dependence on signals, which is not conducive to the differentiation of false IMF components (Bao et al., 2009). Mutual Information (represented by the symbol IMI) can accurately calculate the correlation between the IMF component and the original signal, and has certain advantages in distinguishing the false IMF component.

Therefore, our paper uses IMI to select IMF components. IMI describes the degree of correlation between two random variables, and the amount of common information between two variables can be measured by IMI. The larger the IMI, the more common information between variables, and the stronger the correlation. For the *i*th IMF component *c_i_(t)* of the signal and the original signal *x(t)*, the IMI between them is defined as:


(5) $$I_{MI}\left(c_{i};x\right)=-\Sigma p\left(c_{i};x\right)\log\frac{p\left(c_{i};x\right)}{p\left(c_{i}\right)p\left(x\right)}$$

where, *p(c_i_)* and *p(x)* are the marginal probability distributions of the *i*th IMF component *c_i_(t)* and the original signal *x(t)* respectively; *p(c_i_,x)* is the joint probability distribution of the *i*th IMF component *c_i_(t)* and the original signal *x(t)*.

After the decomposition of CEEMDAN and the selection of IMI, HT is used to calculate the instantaneous frequency and amplitude for IMF. The impulse response of HT is:


(6) $$h\left(t\right)=\frac{1}{\pi t}$$

The HT expression of IMF is:


(7) H(IMF(t))=h(t)∗IMF(t)

where, H(∙) is the function of HT, * is the convolution. Then:


(8) $$Z\left(t\right)=IMF\left(t\right)+jH\left(IMF\left(t\right)\right)=\alpha \left(t\right)e^{j\theta \left(t\right)}$$

where


(9) $$a\left(t\right)=\sqrt{IMF^{2}\left(t\right)+H^{2}\left(IMF\left(t\right)\right)}$$

(10) $$\theta \left(t\right)=\arctan\frac{H\left(\mathrm{IMF}\left(t\right)\right)}{\mathrm{IMF}\left(t\right)}$$

Therefore, the instantaneous frequency can be expressed as:


(11) $$F\left(t\right)=\frac{d\theta \left(t\right)}{dt}$$

*F(t)* and *a(t)* are the instantaneous frequency and amplitude of IMF, respectively. Based on the instantaneous frequency value of IMF, IMFs with different instantaneous frequency values can be obtained by selecting different sampling frequency for each electrode data and decomposing them. The instantaneous frequency value is adjusted to the frequency range of the above 5 frequency bands, which can obtain the corresponding 5 frequency bands. In addition, the time information can be saved after microstate extraction.

#### 2.1.2. Microstate analysis

The core of microstate analysis is to segment EEG data into microstates by using clustering algorithms. The well-established standardized procedures in EEGLAB (Poulsen et al., 2020) are used for the microstate analysis. The specific processes are as follows:

(5) The quantized scalar values for electric potentials across EEG electrodes also known as Global Field Potential (GFP) are computed for EEG: the standard deviation of voltage values at all electrodes of a topographic map at a time, which is used to describe the strength of the electric field of a topographic map. The formula is as follows:

(12) $$GFP\left(t\right)=\sqrt{\frac{1}{k}\left(\overset{K}{\underset{i=1}{\sum }}{\left[V_{i}\left(t\right)-V_{mean}\left(t\right)\right]}^{2}\right)}$$

where, K is the number of channels, Vi(t) is the voltage and potential at the ith electrode, and Vmean(t) is the instantaneous average potential between electrodes.

GFP represents the intensity of the electric field on the brain at every moment. It is usually used to measure the total response of the brain to the event or to represent the rapid changes in brain activity. The local maximum of its curve represents the moment of the strongest field intensity and the highest signal-to-noise ratio. Therefore, using the topographic map at the peak of GFP to represent other surrounding topographic maps for analysis is an effective method to improve the microstate signal-to-noise ratio and reduce the amount of computation (Murray et al., 2008). At the same time, it is also found that the topographic map at the peak of GFP is similar to the surrounding one, while the similarity at the valley is low (Pascual-Marqui and Michel, 1995; Thomas et al., 2002; Walther, 2005), which means that the transition from one topographic map to another is completed at the negative peak of GFP.

(6) The modified k-means clustering algorithm is used for cluster analysis (Murray et al., 2008), and EEG data is clustered into n microstates. Clustering model is as follows:

(13) $$x_{n}=Az_{n}+\varepsilon_{n}$$

where, *x_n_* is the EEG signal sampled for the nth time, 1 ≤ *n* ≤ *N*, *N* is the number of time sample; *A*∈*R^C × K^* is the topographic map of clustering, C is the number of channels, and K is the number of clustering (the number of microstate class). *z_n_*∈*R^K × N^* is the activation state of the microstate at the nth sampling; *ε_n_* is EEG signal noise sampled at the *n*th time.

(7) Global Explained Variance (GEV) and Cross-Validation criterion (Poulsen et al., 2020; CV) are calculated to evaluate the fitness of microstates and determine the optimal number of microstates.

GEV is an index to measure the similarity between each EEG sample and its assigned microstate, so the higher the GEV value, the better the result. The formula is as follows:


(14) $$GEV_{n}=\frac{\left(corr\left(x_{n},a_{l_{n}}\right)\right)\cdot GFP_{n}}{\sum_{n=1}^{N}GFP_{n}^{2}}$$

where, GFPn is the global field potential and the standard deviation of all EEG electrodes sampled at the nth time.

The value of CV is related to residual noise, so a smaller value of CV should be obtained. The calculating formula is as follows:


(15) $$CV={\hat{\sigma }}^{2}\cdot {\left(\frac{C-1}{C-K-1}\right)}^{2}$$

(16) $${\hat{\sigma }}^{2}=\frac{\sum_{n=1}^{N}x_{n}^{T}x_{n}-{\left(a_{l_{n}}^{T}x_{n}\right)}^{2}}{N\left(C-1\right)}$$

where, $\hat{\sigma }$ is the estimator of the residual noise variance.

(8) After matching the extracted microstates to the EEG signals of the subjects, the EEG microstate parameters between the two tasks at each frequency band are calculated respectively:

① Mean Duration (MD): the mean duration of time that one microstate keeps stable.
② Time Coverage Ratio (TCR): percentage in time coverage of one type of microstate.
③ Occurrence Per Second (OPS): frequency of occurrence of one microstate.
④ Global Explained Variance (GEV): an index to measure the similarity between each EEG sample and its assigned microstate.

### 2.2. Data and pre-processing

The dataset used in this paper is derived from a novel cognition-guided neurofeedback BCI dataset on nicotine addiction, which includes smoking subjects performing two cognitively guided tasks at a sampling frequency of 250 Hz (Bu et al., 2021). The cognitively guided task of the dataset is to record EEG data by allowing subjects to focus on the smoking-related pictures (e.g., holding a cigarette in hand) and paired neutral pictures (e.g., holding a pencil in hand). The EEG data of smoking-related pictures and neutral pictures on each subject were recorded in six groups, respectively.

In this study, EEG data of 20 subjects were selected from this dataset, including 120 groups of EEG data in smoking-related pictures (smoke) and 120 groups of EEG data in neutral pictures (neutral). In the pre-processing step, the original EEG signals were filtered to 0.1–40 Hz to remove noise and other interference signals. At the same time, the eye electrodes were removed, and the corresponding potentials of 45 electrodes (F7, F5, F3, F1, FZ, F2, F4, F6, F8, FT7, FC5, FC3, FC1, FCZ, FC2, FC4, FC6, FT8, T7, C5, C3, C1, CZ, C2, C4, C6, T8, TP7, CP5, CP3, CP1, CPZ, CP2, CP4, CP6, TP8, P7, P5, P3, P1, PZ, P2, P4, P6, P8) were selected for evaluation.

By using the CEEMDAN and instantaneous frequency model in the improved HHT method, our experiment divided the addiction EEG data with two different tasks into five frequency bands including delta band (0.5–4 Hz), theta band (4–8 Hz), alpha band (8–12 Hz), beta band (12–30 Hz), and gamma band (30–40 Hz). Then, we analyzed and compared the microstate parameters at each frequency band.

## 3. Results

### 3.1. Full band microstates

**Figure 1:**
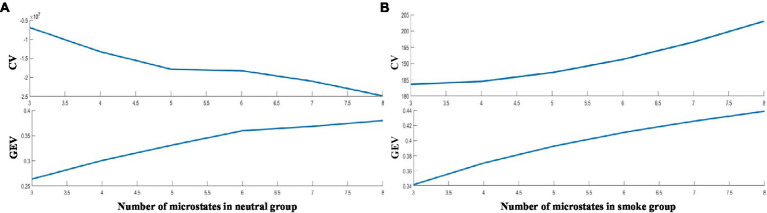
The effective number of microstates based on the fitness.

**Figure 2:**
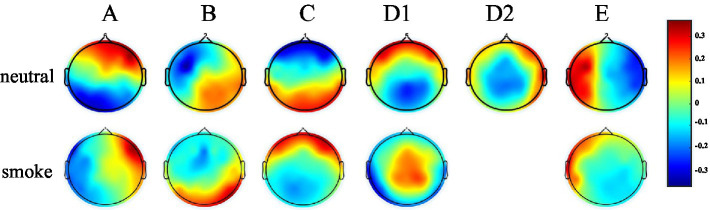
EEG microstates on two response tasks, the number in neutral group is 6, the number in smoke group is 5.

According to GEV and CV, the difference is the largest when the number of EEG microstates in neutral group is 6 and smoke group is 5 in Figure 1. Therefore, we selected 6 microstates in neutral group and 5 in smoke group, which is shown in Figure 2. Microstates A, B, and C in neutral group and smoke group are similar to the classic microstates. However, microstate D, with positive and negative voltage located in the frontal central region (ignoring polarity), is related to attention network and sleep (Delorme and Makeig, 2004), which is split into microstates D1 and D2 in neutral group, but does not split in smoke group. In addition, an additional microstate E is generated in both groups.

### 3.2. Time-frequency analysis

**Figure 3:**
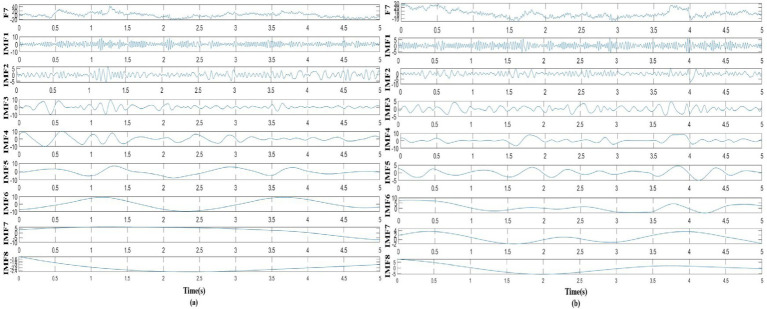
Taking channel F7 as an example, the EEG signal was decomposed into IMFs, where **(A)** decomposed signal in neutral group, **(B)** decomposed signal in smoke group.

**Figure 4:**
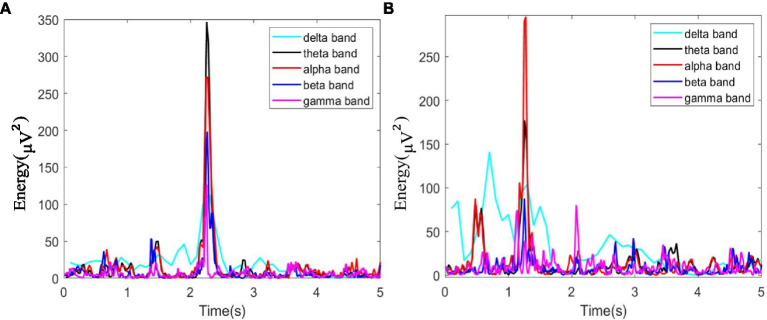
Energy diagram at each band of F7 channel within 5 s, where **(A)** neutral group; **(B)** smoke group.

**Figure 5:**
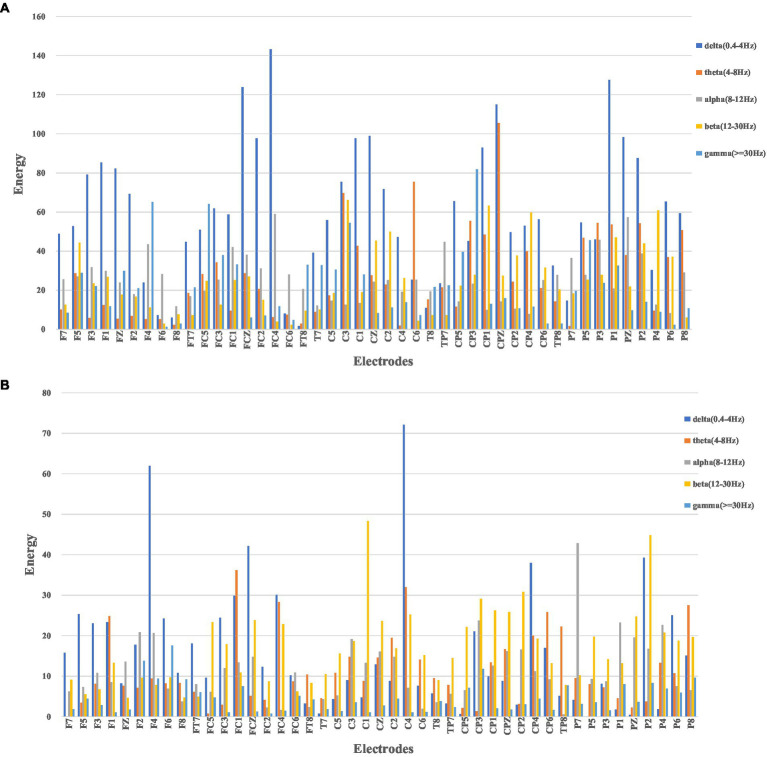
Sub-band energies across 45 electrodes, where **(A)** neutral group; **(B)** smoke group.

In this study, as mentioned above, instantaneous parameters were extracted to provide an insight into the local variations in the spectral domain of EEG data. For each subject, and for each channel, the EEG data were decomposed into a set of IMFs using the CEEMDAN algorithm, which is followed by the estimation of instantaneous amplitudes and instantaneous frequency using HT. As an example, Figure 3 shows the decomposed IMFs for channel F7 of EEG data from a representative subject. Figure 4 shows the corresponding energies at each band. It should be noted that the whole-time length of 1 min is used for decomposition and for a better display only 5 s data are shown. Figure 5 shows the sub-band energies across 45 electrodes for one time instance.

### 3.3. Sub-band microstates and statistical analysis

**Figure 6:**
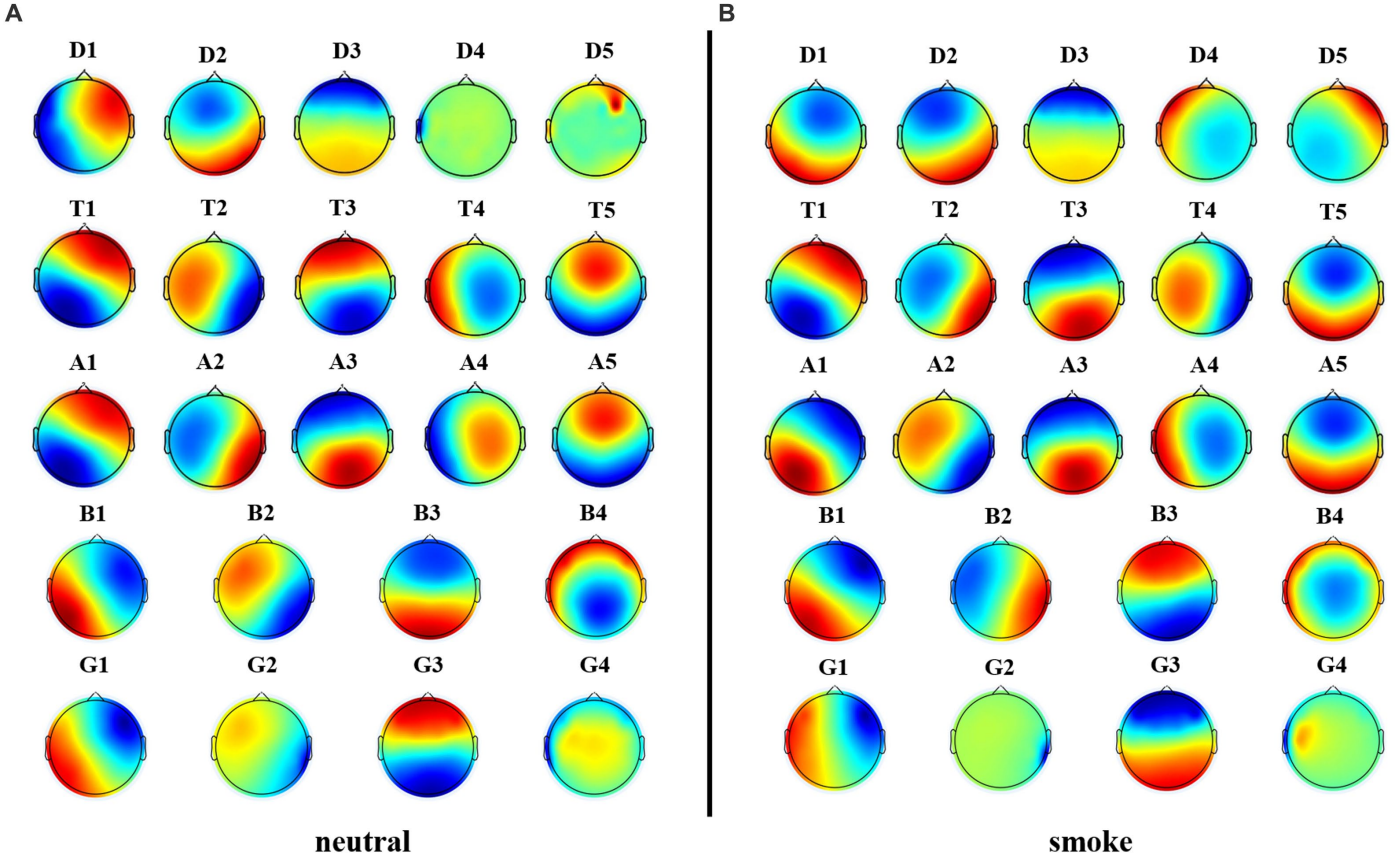
Microstate topographic maps at each frequency band, i.e., delta band (D1, D2, D3, D4, D5), theta band (T1, T2, T3, T4, T5), alpha band (A1, A2, A3, A4, A5), beta band (B1, B2, B3, B4), and gamma band (G1, G2, G3,G4).

We performed microstate analysis at each EEG frequency band obtained by improved HHT method. According to GEV and CV, the optimal number of microstate at delta, alpha and theta bands is 5, while the optimal number of microstate at beta and gamma bands is 4. Figure 6 shows each band microstate topographic maps obtained by improved HHT method.

Microstate parameters at each frequency band under the improved HHT method were calculated, including MD, OPS, TCR, and GEV. The results are shown in Table 1. Multi-way ANOVA was performed for microstate parameters at each frequency band. We find significant class × group interactions for all microstate parameters: ① delta band: MD (*F* = 120.98, *p* < 0.001, η^2^ = 347164.93), OPS (*F* = 702.13, *p* < 0.001, η^2^ = 140.94), TCR (*F* = 551.46, *p* < 0.001, η^2^ = 5.55), GEV (*F* = 305.58, *p* < 0.001, η^2^ = 4.78); ② alpha band: GEV (*F* = 9.98, *p* < 0.001, η^2^ = 0.047); ③ beta band: TCR (*F* = 3.42, *p* = 0.017, η^2^ = 0.04), GEV (*F* = 25.45, *p* < 0.001, η^2^ = 0.16); Then, we performed separate one-way ANOVA for each microstate parameter at delta, alpha and beta bands between neutral and smoke group. The results are shown in “Supplementary Tables A.” These follow-up tests reveals significant between group differences for band microstates: ① At delta band, the OPS, TCR and GEV of microstate D5 in neutral group are higher than those in smoke group (OPS_neutral_ = 1.59 ± 0.30, OPS_smoke_ = 1.44 ± 0.20; TCR_neutral_ = 0.22 ± 0.11, TCR_smoke_ = 0.18 ± 0.03; GEV_neutral_ = 7.75 ± 2.49, GEV_smoke_ = 6.64 ± 1.81); ② At alpha band, the OPS, TCR and GEV of microstate A2 in neutral group are lower than those in smoke group (OPS_neutral_ = 2.03 ± 0.22, OPS_smoke_ = 2.14 ± 0.15; TCR_neutral_ = 0.21 ± 0.03, TCR_smoke_ = 0.22 ± 0.02; GEV_neutral_ = 7.62 ± 1.65, GEV_smoke_ = 8.62 ± 1.65); ③ At beta band, the TCR and GEV of microstate B2 in neutral group are lower than those in smoke group (TCR_neutral_ = 0.25 ± 0.04, TCR_smoke_ = 0.27 ± 0.02; GEV_neutral_ = 7.47 ± 1.89, GEV_smoke_ = 8.67 ± 3.53).

**Table 1:** Microstate parameters at each frequency band under the improved HHT method.

Subjects	Microstates	MD		OPS		TCR		GEV	
		neutral	smoke	neutral	smoke	neutral	smoke	neutral	smoke
Delta	D1	127.66 ± 10.97	131.01 ± 14.50	1.62 ± 0.39	1.63 ± 0.40	0.21 ± 0.06	0.22 ± 0.07	19.30 ± 6.60	20.33 ± 9.49
	D2	125.50 ± 7.63	127.18 ± 7.83	1.49 ± 0.49	1.68 ± 0.20	0.19 ± 0.07	0.21 ± 0.03	11.77 ± 2.33	12.54 ± 3.79
	D3	123.63 ± 9.19	126.21 ± 6.37	1.45 ± 0.42	1.59 ± 0.16	0.18 ± 0.06	0.20 ± 0.03	9.55 ± 1.90	9.26 ± 1.93
	D4	127.82 ± 23.78	125.16 ± 7.34	1.57 ± 0.28	1.53 ± 0.20	0.21 ± 0.09	0.19 ± 0.03	9.19 ± 3.66	8.27 ± 1.96
	D5	130.38 ± 34.43	121.22 ± 5.64	1.59 ± 0.30	1.44 ± 0.20	0.22 ± 0.11	0.18 ± 0.03	7.75 ± 2.49	6.64 ± 1.81
Theta	T1	123.31 ± 7.28	122.23 ± 8.37	1.95 ± 0.17	1.89 ± 0.16	0.24 ± 0.03	0.23 ± 0.03	10.00 ± 1.93	10.04 ± 2.32
	T2	117.21 ± 5.98	119.86 ± 6.50	1.85 ± 0.11	1.82 ± 0.14	0.22 ± 0.02	0.22 ± 0.02	8.41 ± 1.44	8.45 ± 1.61
	T3	115.51 ± 4.88	118.03 ± 6.38	1.74 ± 0.12	1.74 ± 0.11	0.20 ± 0.02	0.21 ± 0.02	7.37 ± 1.37	7.59 ± 1.19
	T4	110.36 ± 5.43	112.61 ± 5.81	1.59 ± 0.13	1.64 ± 0.13	0.18 ± 0.02	0.18 ± 0.02	6.23 ± 0.96	6.40 ± 0.79
	T5	109.74 ± 5.89	106.56 ± 4.40	1.49 ± 0.17	1.51 ± 0.15	0.16 ± 0.02	0.16 ± 0.02	5.29 ± 0.84	5.27 ± 0.89
Alpha	A1	108.60 ± 6.93	105.67 ± 5.46	2.20 ± 0.20	2.21 ± 0.17	0.24 ± 0.03	0.23 ± 0.02	9.64 ± 2.22	9.56 ± 1.50
	A2	101.13 ± 6.60	104.07 ± 3.83	2.03 ± 0.22	2.14 ± 0.15	0.21 ± 0.03	0.22 ± 0.02	7.62 ± 1.65	8.62 ± 1.65
	A3	100.27 ± 6.50	102.84 ± 5.59	1.96 ± 0.19	2.01 ± 0.17	0.20 ± 0.03	0.21 ± 0.02	7.18 ± 1.90	7.41 ± 1.47
	A4	97.52 ± 4.45	97.24 ± 6.64	1.85 ± 0.18	1.79 ± 0.15	0.18 ± 0.02	0.17 ± 0.22	6.13 ± 0.82	5.97 ± 1.03
	A5	96.73 ± 5.82	95.94 ± 4.41	1.81 ± 0.22	1.70 ± 0.15	0.18 ± 0.03	0.16 ± 0.02	5.69 ± 1.69	5.16 ± 0.90
Beta	B1	87.81 ± 6.92	83.90 ± 16.97	3.39 ± 0.16	3.88 ± 2.70	0.30 ± 0.03	0.29 ± 0.03	9.68 ± 3.47	9.48 ± 3.55
	B2	80.62 ± 5.13	80.14 ± 14.76	3.11 ± 0.45	3.81 ± 2.58	0.25 ± 0.04	0.27 ± 0.02	7.47 ± 1.89	8.67 ± 3.53
	B3	80.50 ± 3.65	76.48 ± 14.88	1.62 ± 0.39	1.63 ± 0.40	0.25 ± 0.03	0.23 ± 0.03	6.60 ± 1.17	6.35 ± 1.39
	B4	76.90 ± 5.42	76.24 ± 15.44	1.49 ± 0.49	1.68 ± 0.20	0.20 ± 0.03	0.21 ± 0.03	4.91 ± 1.18	5.25 ± 1.33
Gamma	G1	120.30 ± 52.82	138.77 ± 102.42	1.45 ± 0.42	1.59 ± 0.16	0.30 ± 0.07	0.31 ± 0.08	10.09 ± 5.82	10.83 ± 7.82
	G2	106.48 ± 53.52	93.68 ± 8.23	1.57 ± 0.28	1.53 ± 0.20	0.27 ± 0.05	0.26 ± 0.04	7.18 ± 2.88	6.52 ± 1.84
	G3	90.54 ± 12.54	87.53 ± 20.00	1.59 ± 0.30	1.44 ± 0.20	0.22 ± 0.05	0.22 ± 0.08	4.68 ± 1.28	4.71 ± 2.10
	G4	101.80 ± 57.41	94.04 ± 26.97	1.95 ± 0.17	1.89 ± 0.16	0.21 ± 0.05	0.21 ± 0.06	3.98 ± 1.17	4.51 ± 1.64

### 3.4. Classification and recognition on microstate parameters

According to the results in section 3.3, microstate parameters with significant differences between the two tasks were selected as features, including MD, OPS, TCR and GEV at delta, alpha, and beta band, which were performed for classification under Gaussian kernel SVM classifier.

The results are shown in Table 2. The classification effect is optimal at delta band. The microstate D1 has the highest classification accuracy (92%), sensitivity (94%) and specificity (91%). Other microstates at delta band also have better classification results. Then, microstate A2 and A3 at alpha band also have good classification effect, with the highest accuracy of 78%, sensitivity of 75% and specificity of 87%. Furthermore, microstate B1 and B2 at beta band have general classification effect, with accuracy of 73%, sensitivity of 90% and specificity of 84%.

**Table 2:** Classification results of microstates at delta, alpha, and beta bands by using improved HHT-Microstate.

Subjects	Microstates	Accuracy (%)	Sensitivity (%)	Specificity (%)
Delta [0.1–4 Hz]	D1	92.86	94.29	91.43
	D2	90.35	90.03	90.29
	D3	83.33	68.89	91.11
	D4	87.88	86.02	88.14
	D5	83.34	66.67	91.36
Alpha [8–12 Hz]	A1	69.44	75.08	63.89
	A2	78.30	73.58	83.02
	A3	71.28	46.81	87.74
	A4	56.71	64.86	48.16
	A5	61.86	58.28	64.22
Beta [12–30 Hz]	B1	70.19	55.77	84.62
	B2	73.47	90.88	53.06
	B3	61.02	61.02	61.02
	B4	63.64	70.45	56.82

**Figure 7:**
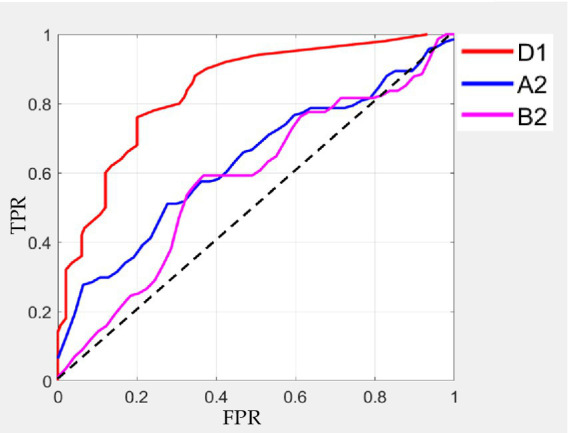
ROC curves of classification on microstate D1, A2, and B2..

Besides, selecting the microstates D1, A2, and B2 with the best classification result, we also plot the ROC curve, which shows that D1 has the best results in classification. The specific result is shown in Figure 7.

### 3.5. Comparing with other methods in nicotine addiction detection

Some EEG analysis methods which similar to the improved HHT-Microstate were also employed to analyze EEG data of these nicotine addiction subjects. Previous studies have greatly improved the decoding accuracy of EEG by calculating the spatial feature of the Riemann distance in the EEG of motion imagination at frequency bands (Qu et al., 2022). And in the traditional microstate analysis, the Finite Impulse Response (FIR) filter in EEGLAB (Michel and Koenig, 2017) was employed to filter EEG data according to the frequency band range. Therefore, microstates and Riemann distance were calculated from EEG signal at each frequency band filtering by FIR and HHT. The result of analysis and comparison according to these methods are as follows.

**Figure 8:**
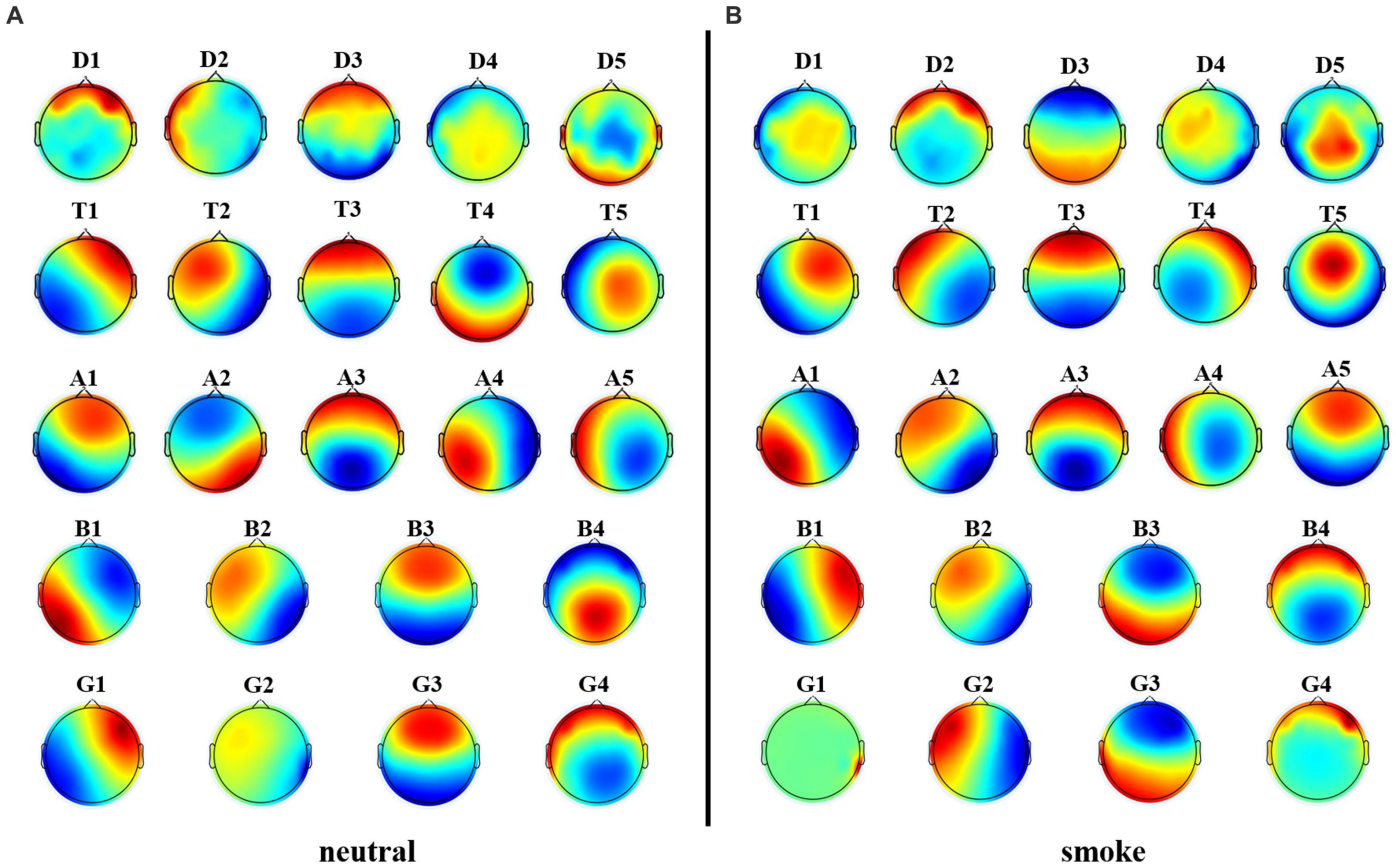
Microstate topographic maps at each frequency band, i.e., delta band (D1, D2, D3, D4, D5), theta band (T1, T2, T3, T4, T5), alpha band (A1, A2, A3, A4, A5), beta band (B1, B2, B3, B4), and gamma band (G1, G2, G3,G4).

Figure 8 shows each band microstate topographic maps obtained by FIR method. For further comparison with the improved HHT-Microstate, similarity index (Kingsley and Sethukarasi, 2023) was calculated for each single-band and full-band microstate topographic maps, respectively, for the purpose of corresponding comparison.

**Figure 9:**
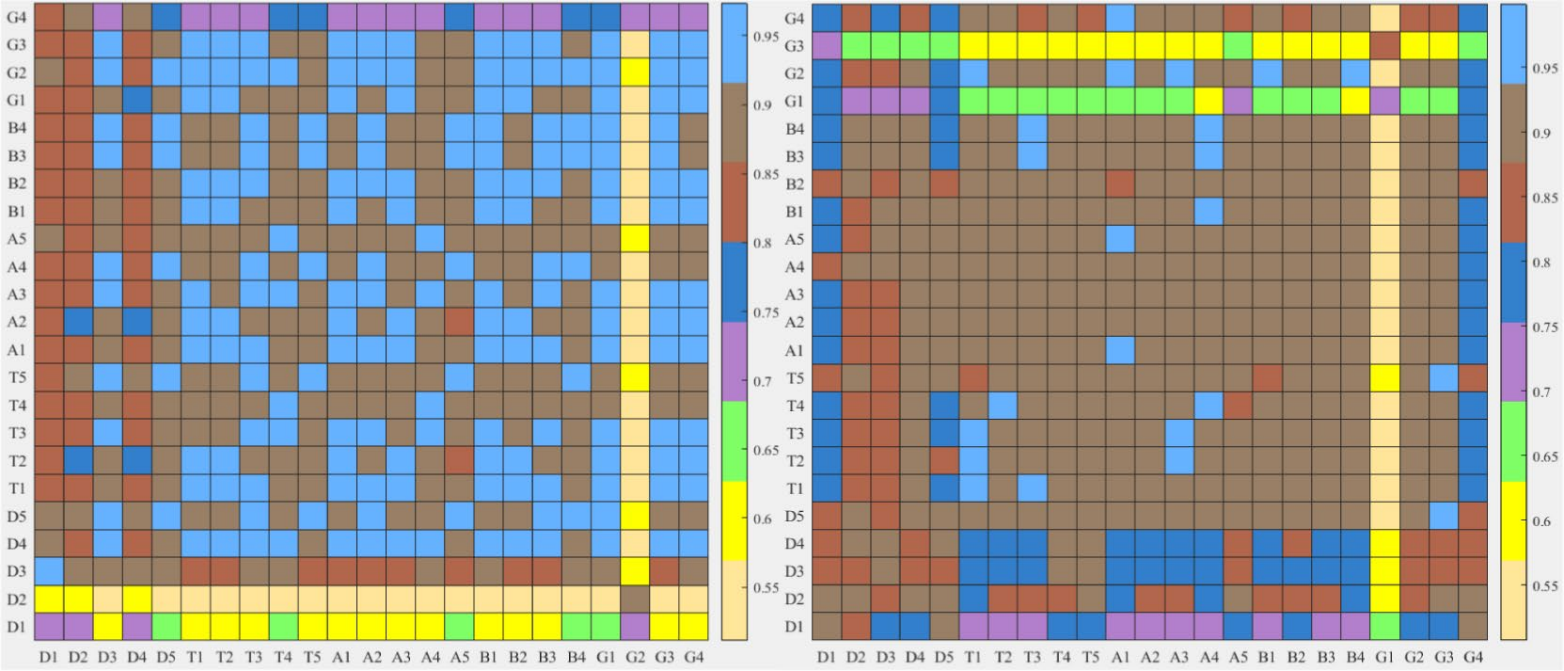
After frequency band division by the two methods, the similarity index hot plot of all microstate topographic maps at each EEG frequency band between two kinds of different tasks is extracted. The horizontal axis is EEG microstate of the neutral group and the vertical axis is EEG microstate of the smoke group.

Firstly, the difference index of each EEG band topographic maps between the two types of tasks was compared. The results in Figure 9 show that the improved HHT method provides more variability among the topographic maps at each frequency band.

Secondly, the permutation test was conducted for the similarity of the two topographic maps (Koenig et al., 1999). Table 3 shows the permutation test results of the similarity index among topographic maps. The difference of the test results under the improved HHT method is mainly reflected in the microstate A2 and A5 at alpha band and the microstate B4 at beta band.

**Table 3:** The permutation test results of the similarity index between the full-band and single-band microstate topographic map of subjects’ EEG.

Microstates		A		B		C		D		E	
Bands		Improved HHT	FIR	Improved HHT	FIR	Improved HHT	FIR	Improved HHT	FIR	Improved HHT	FIR
Delta	D1	0.31	0.85	0.28	0.86	0.34	0.88^*^	0.29	0.83	0.35	0.86^*^
	D2	0.25	0.86	0.22	0.86	0.27	0.88	0.23	0.83	0.34	0.85
	D3	0.23	0.86	0.20	0.86	0.24	0.88	0.22	0.84	0.32	0.86
	D4	0.30	0.87	0.38	0.87	0.39	0.89	0.38	0.84	0.35	0.87
	D5	0.37	0.87	0.35	0.87	0.32	0.89	0.37	0.84	0.38	0.87
Theta	T1	0.25	0.79	0.23^*^	0.79	0.27	0.85	0.23^*^	0.76	0.34	0.92
	T2	0.13	0.80	0.13	0.80	0.16	0.86	0.16	0.77	0.22	0.92
	T3	0.28	0.79	0.29	0.79	0.26	0.85	0.30	0.76	0.22	0.92
	T4	0.30	0.81	0.24	0.80	0.39	0.86	0.25	0.77	0.42	0.91
	T5	0.20	0.82	0.15	0.81	0.24	0.87	0.19	0.78	0.29	0.92
Alpha	A1	0.47	0.80	0.47	0.79	0.47	0.85	0.48	0.77	0.33	0.92
	A2	0.04^*^	0.80	0.04	0.79	0.04*	0.85	0.05^*^	0.76	0.04^*^	0.90
	A3	0.30	0.81	0.34	0.80	0.37	0.86	0.32	0.77	0.35	0.92
	A4	0.29	0.80	0.30	0.79	0.29	0.85	0.32	0.77	0.28	0.91
	A5	0.21^*^	0.80	0.19^*^	0.80	0.17^*^	0.86	0.20^*^	0.77	0.11^*^	0.91
Beta	B1	0.18	0.79	0.19	0.80	0.20	0.83	0.14	0.77	——	——
	B2	0.17	0.79	0.17	0.80	0.16	0.82	0.16	0.77	——	——
	B3	0.32	0.80	0.26	0.80	0.23	0.85	0.24	0.77	——	——
	B4	0.50^*^	0.81	0.04^*^	0.81	0.06^*^	0.85	0.04^*^	0.78	——	——
Gamma	G1	0.16	0.76	0.25	0.79	0.21	0.78	0.21^*^	0.76	——	——
	G2	0.22	0.76	0.34	0.80	0.30	0.78	0.28	0.77	——	——
	G3	0.32	0.76	0.31	0.79	0.21	0.78	0.11	0.76	——	——
	G4	0.40	0.80	0.37	0.81	0.27	0.83	0.16	0.78	——	——

The results with statistically significant differences are indicated by asterisks (*).

In addition, the GEV under the two methods were calculated, respectively. The GEV under the improved HHT method is higher than the traditional filtering method, which are shown in Table 4.

**Table 4:** GEV for all microstates at each frequency band under improved HHT method and traditional filtering method.

EEG Data	Methods	Delta	Theta	Alpha	Beta	Gamma
Neutral	Improved HHT	55.17	37.93	36.77	27.10	21.15
	FIR	31.79	33.20	34.89	24.50	33.19
Smoke	Improved HHT	55.14	38.29	37.15	27.57	22.87
	FIR	32.49	32.8	34.99	28.39	34.21

Furthermore, microstate parameters and Riemann distance at each frequency band calculated under the FIR method were shown in “Supplementary Tables B,” which include MD, OPS, TCR and GEV for microstate, model of AIRM, Stein, Jeffery and LogED for Riemann distance. Multi-way ANOVA was performed for each parameter at each frequency band. We do not find significant class × group interactions for all parameters. We also did one-way ANOVA for each microstate parameter and Riemann distance at delta, alpha and beta bands between neutral and smoke group. The results were shown in “Supplementary Tables C.” Only few parameters have significant difference.

Finally, we also select microstate parameters and Riemann distance at delta, alpha and beta bands between the two tasks as features, which were performed for classification under Gaussian kernel SVM classifier.

The results are shown in Tables 5–7. The optimal effect of classification for microstate is A4 at alpha band, which has the highest classification accuracy (87%), sensitivity (88%) and specificity (86%), and for Riemann distance it is beta band, which has the highest classification accuracy (71%), sensitivity (67%) and specificity (75%). The effect of classification for FIR-Microstate and FIR-Riemann is inferior to improved HHT-Microstate.

**Table 5:** Classification results of microstates at delta, alpha, and beta bands by using FIR-Microstate.

Subjects	Microstates	Accuracy (%)	Sensitivity (%)	Specificity (%)
Delta [0.1–4 Hz]	D1	64.44	62.22	66.67
	D2	66.67	68.89	64.44
	D3	62.22	93.33	31.11
	D4	77.78	86.67	31.11
	D5	77.78	73.33	82.22
Alpha [8–12 Hz]	A1	54.00	60.00	48.00
	A2	65.00	52.00	78.00
	A3	63.00	62.00	64.00
	A4	87.00	88.00	86.00
	A5	79.00	64.00	94.00
Beta [12–30 Hz]	B1	65.91	81.82	50.00
	B2	64.78	72.73	56.82
	B3	64.77	72.73	56.82
	B4	65.91	77.27	54.55

**Table 6:** Classification results of Riemann distance at delta, alpha, and beta bands by using HHT-Riemann.

Bands	Accuracy (%)	Sensitivity (%)	Specificity (%)
Delta	62.22	55.56	68.89
Alpha	57.78	64.44	51.11
Beta	64.29	71.43	57.14

**Table 7:** Classification results of Riemann distance at delta, alpha, and beta bands by using FIR-Riemann.

Bands	Accuracy (%)	Sensitivity (%)	Specificity (%)
Delta	60.00	51.11	68.89
Alpha	57.45	57.45	57.45
Beta	71.43	67.35	75.51

## 4. Discussion

As a category of mental illness, substance addiction is a cause of avoidable morbidity and mortality around the world. Nicotine addiction is the most widely distributed and the most numerous substance addiction type. According to relevant studies, nicotine addicts are different from non-addicts in cognitive function, sleep structure and smoking cue response. Therefore, many studies mainly carry out on the mechanism of addiction and intervention methods, which have great potential clinical benefits for the intervention and treatment of substance addiction. Spontaneous EEG signal, modulated by cognitive and sensory processing (Samaha et al., 2022), fluctuates in milliseconds and explains the transient brain functional states. Therefore, it is necessary to further explore the brain mechanism of smoking cue response and find effective markers of smoking cue response as targets for addiction detection and intervention.

In order to determine the fluctuation dynamics of brain neural sources in the time domain, Lehman (Lehmann et al., 1987; Lehmann, 1994) proposed the method of EEG microstate analysis which quantified the spatial distribution of nerve potentials among scalp electrodes at each time, reflected the sum of instantaneous activity of brain neutral clusters with fewer microstate topographic maps, and examined the functional activity network of the brain (Lehmann et al., 1987). It is the best choice for time domain analysis. On this basis, Ehtasham et al. (2019) used the HHT method to transform the time-domain data into the spectral domain data, which retained its instantaneous characteristics, constructed the correlation between microstate and spectral features, and proved that this method was superior to the traditional filtering method through experimental comparison. Therefore, in this paper, an improved HHT method and FIR method were used, respectively, for frequency band decomposition and microstate analysis on EEG of nicotine addiction under two types of different tasks. Besides, microstate parameters with significant differences after improved HHT decomposition were used as features to classify and detect nicotine addiction.

### 4.1. Comparison of microstate parameters at each frequency band

Since cue response plays an important role in the psychological cognition, withdrawal and relapse of nicotine addictions, many experiments have been conducted to study the different cues provided by nicotine addicts, which include the cues related to neutral control and cigarettes to different degrees. Through different cue feedback, it is found that cigarette-related cues caused higher levels of de-alpha synchronization (Cui et al., 2013), and theta band in frontal lobe shows strong network coherence in smoking cues (Shinan, 2020). At the same time, studies have shown that there are significant differences in microstate parameters between addicts and non-addicts, and better identification and detection can be achieved under the SVM optimized by genetic algorithm (Peng, 2019). Thus, combined with previous studies, whether there are significant differences in microstate parameters at different frequency bands and whether accurate identification and detection can be achieved in the face of different smoking cues is a worth studying problem in the cue response of substance addicts.

Therefore, in our paper, two time-frequency decomposition methods were employed to divide the EEG data of nicotine addicts into five different bands and compared the microstate parameters between two different cue-response tasks at each frequency band to find differences.

The experimental results show that the two groups of full band microstates in Figure 2, microstate D in neutral group split into microstates D1 and D2, while those in smoke group does not split. In addition, an additional microstate E is generated in both tasks groups. Whether this microstate is unique to nicotine addicts needs further research. Then, MD, OPS, TCR and GEV at each frequency band obtained by the improved HHT method are shown in Table 1. According to the result of multi-way ANOVA, we find significant class × group interactions for microstate D5, A2 and B2 at specific delta, alpha and beta bands. However, the study of Ehtasham et al. (2019) found that the optimal number of microstates at each frequency band was 4, and the parameters were similar and consistent at each frequency band, without significant difference through healthy non-addicts. Our experimental results are similar to previous studies that there are significant differences in coherence, power, and energy between addicts and non-addicts at specific EEG bands (Reid et al., 2004; Peng, 2019), which may indirectly indicate that the EEG of nicotine addicts is different at certain frequency bands. Therefore, the difference of EEG microstate parameters at specific frequency bands can be used to detect substance addiction. Furthermore, based on the results of one-way ANOVA for each microstate parameter between neutral and smoke group at delta, alpha and beta bands, D5_OPS_, D5_TCR_ and D5_GEV_ in neutral group at delta band are higher than those in smoke group, A2_OPS_, A2_TCR_ and A2_GEV_ in neutral group at alpha band are lower than those in smoke group and B2_TCR_, B2_GEV_ in neutral group at beta band are higher than those in smoke group. We can distinguish and detect nicotine addiction with different cue responses mainly by these microstate parameters at these frequency bands.

### 4.2. Detection of nicotine addiction by improved HHT-microstate method

Previous studies have used microstate correlation parameters to classify and detect different diseases or tasks and have achieved better identification and detection effect. For example, for heroin addicts, the features of EEG microstate parameters and negative peak of microstate duration were used to classify, with the accuracy of 72% (Peng, 2019). At the same time, there are different spatial microstates between patients with high and normal cranial pressure. Microstate parameters were used to classify patients with high and low cranial pressure, which can obtain the highest classification accuracy (87%; Shuaiyang, 2021). In this paper, microstate parameters with different frequency bands under different cue-response tasks were selected as features to the Gaussian kernel SVM classifier for classification and detection. The microstate parameters at delta band, alpha band and beta band were used to classify substance addiction. The microstate D1 at delta band has the highest classification accuracy (92%), sensitivity (94%), and specificity (91%), the microstate A2 at alpha band and microstate B2 at beta band also have better classification result. At the same time, the microstates with the best classification result at each band were selected and plot the ROC curves, which also mainly reflected the best result on microstate D1 and the better result on microstate A2 and B2 by evaluating the AUC under each curve. Therefore, microstate parameters at delta and alpha bands are promising for the identification and detection of nicotine addiction.

### 4.3. Comparison of analysis result between the improved HHT-microstate and other methods

In order to further prove the intrinsic superiority of the improved HHT-Microstate method, this experiment compared the improved HHT-Microstate method with other similar EEG analysis methods, including the frequency band microstates and frequency band Riemann distance extracted by FIR filtering methods, and conducted corresponding statistical analysis and classification detection, respectively. According to the comparison results in section 3.5, it is found that there were significant differences on the similarity of microstate topographic maps, statistical analysis and classification results of parameters between different methods.

Firstly, according to Figures 6, 8, it could obviously observe that there were significant differences between the two methods for each EEG microstate topographic map at delta and gamma bands in neutral and smoke group, and there were also some other significant differences in the microstate topographic maps at other bands. The similarity index among all topographic maps at each EEG frequency band of the two groups obtained by the improved HHT and FIR is shown in Figure 9, in which the improved HHT method has a lot of variability among the band topographic maps. According to the permutation test in Table 3, the similarity of improved HHT-Microstate method at alpha and beta bands is significantly different. However, the FIR method does not detect these differences. In addition, the GEV under the improved HHT-Microstate method is higher than the FIR method.

Then, the same multi-way ANOVA as improved HHT-Microstate method was performed on the FIR band microstate parameters and Riemann distance, however, there were no significant interaction. At the same time, only a few parameters of each feature were significantly different under the one-way ANOVA between neutral and smoke group. For more accurate verification, we also chose feature parameters consistent with improved HHT-Microstate method for classification detection, which means that microstate parameters and Riemann distance at delta, alpha and beta band were selected as features for classification. According to the results in Tables 5–7, it is found that the result of classification for microstate is A4 at alpha band, which has the best accuracy (87%), sensitivity (88%) and specificity (86%), and for Riemann distance is beta band, which has the best accuracy (71%), sensitivity (67%) and specificity (75%). Thus, the effect of classification for the improved HHT-Microstate is better than FIR-Microstate, HHT-Riemann and FIR-Riemann methods, which means that the improved HHT-Microstate method is more suitable to represent the characteristics of EEG microstates and more representative than other methods in describing the dynamic characteristics of EEG.

### 4.4. Expectation

As a widespread medical and social problem in the world, substance addiction causes great harm to the physical health of human and the stability of society. At present, the main treatment methods are physical therapy, drug therapy, psychological therapy and neurofeedback therapy. In recent years, with the development of BCI, EEG research has become a new diagnostic basis and treatment for substance addiction, which includes analyzing and comparing the differences of EEG signals in substance addiction, addiction withdrawal, and healthy controls. In this paper, the improved HHT method was used to divide the frequency band of EEG data and preserve the instantaneous characteristics of the time-domain data in the spectrum domain. At the same time, the EEG microstates of patients with nicotine addiction under different cue-response tasks were compared, and it was found that there were significant differences in the EEG microstates between different tasks.

However, with the advancement of computer technology, an increasing number of computational methods have been applied to brain research, such as Generative Adversarial Network (GAN), which solves the problem of imbalanced medical images (Hu et al., 2020), constructs super-resolution MR Images (You et al., 2022), reconstructs the lost BOLD signal (Yan et al., 2020) and fuse multi-modality medical images (Hu et al., 2021). All of them are latest research results in the brain science field and very enlightening the research of nicotine addiction in this paper. Therefore, our next step is to use these new techniques to analyze EEG signals and discover hidden information, which can be combined with microstate analysis.

Finally, there are individual differences in EEG signals. Increasing the amount of data will help further validate the results of this article, which is also one of our next steps. Besides, the methods of signal process could also be improved, for instance, some other adaptive time-frequency analysis methods (Wacker and Witte., 2011; Hadjileontiadis et al., 2017) can replace the improved HHT method and calculate the corresponding instantaneous frequency or instantaneous amplitude to obtain the unique EEG bands.

## 5. Conclusion

In this paper, we compared the difference of EEG microstates between nicotine addicts by using the improved HHT time-frequency decomposition method. We selected microstate parameters with significant difference as features for classification and got better recognition detection results. These results indicate that the EEG data at frequency bands obtained by the improved HHT method is more suitable to represent the characteristics of EEG signals, and the microstates obtained by this method can be effectively distinguished from the EEG data of nicotine addiction, which means that the improved HHT-Microstate analysis can offer new ideas and insights for the brain research of nicotine addiction and provide more effective methods and basis for the diagnosis and treatment of substance addiction.

## Data availability statement

The original contributions presented in the study are included in the article/Supplementary material, further inquiries can be directed to the corresponding author.

## Author contributions

XX: conceptualization and project administration. JF: methodology. YZ: software. DW: formal analysis. SY: investigation. CW: data curation. JF and RL: writing—original draft preparation. JH: supervision and funding acquisition. All authors contributed to the article and approved the submitted version.

## Funding

This research was funded by the National Nature Science Foundation of China, Yunnan Fundamental Research Projects and Scientific Research Fund of Hanshan Normal University, grant numbers 82060329, 202201AT070108, XY202106, and QD2021218.

## Conflict of interest

The authors declare that the research was conducted in the absence of any commercial or financial relationships that could be construed as a potential conflict of interest.

## Publisher’s note

All claims expressed in this article are solely those of the authors and do not necessarily represent those of their affiliated organizations, or those of the publisher, the editors and the reviewers. Any product that may be evaluated in this article, or claim that may be made by its manufacturer, is not guaranteed or endorsed by the publisher.

## References

[ref1] Andreou C. Faber P. L. Leicht G. Schoettle D. Polomac N. Hanganu-Opatz I. L.. (2014). Resting-state connectivity in the prodromal phase of schizophrenia: insights from EEG microstates. Schizophr. Res. 152, 513–520. doi: 10.1016/j.schres.2013.12.008, PMID: 24389056

[ref2] Arjun K. Alvaro P. L. Christoph M. M. Faranak F. (2014). Microstates in resting-state EEG: current status and future directions. Neurosci. Biobehav. Rev. 49, 105–113. doi: 10.1016/j.neubiorev.2014.12.010 25526823 PMC4305485

[ref3] Arshad J. Qaisar A. Rehman A.-U. Shakir M. Nazir M. K. Rehman A. U.. (2022). Intel-ligent control of robotic arm using brain computer Interface and artificial intelligence. Appl. Sci. 12:10813. doi: 10.3390/app122110813

[ref4] Bao C. Hong H. Li Z. X. Zhu X. (2009). Time-varying system identification using a newly improved HHT algorithm. Comput. Struct. 87, 1611–1623. doi: 10.1016/j.compstruc.2009.08.016

[ref5] Benos J. Kapinas K. (1980). EEG examination in heroin addicts in rehabilitation. Med. Welt 31, 1395–1399. PMID: 7453524

[ref6] Bjork J. M. Gilman J. M. (2014). The effects of acute alcohol administration on the human brain: insights from neuroimaging. Neuropharmacology 84, 101–110. doi: 10.1016/j.neuropharm.2013.07.039, PMID: 23978384 PMC3971012

[ref7] Britz J. Van D. V. D. Michel C. M. (2010). BOLD correlates of EEG topography reveal rapid resting-state network dynamics. Neuroimage 52, 1162–1170. doi: 10.1016/j.neuroimage.2010.02.052, PMID: 20188188

[ref8] Bu J. Liu C. Gou H. Gan H. Zhang X. (2021). A novel cognition-guided Neurofeedback BCI dataset on nicotine addiction. Front. Neurosci. 15:647844. doi: 10.3389/fnins.2021.647844, PMID: 34295217 PMC8290081

[ref9] Chu C. Zhang Z. Wang J. Liu S. Wang F. Sun Y.. (2021). Deep learning reveals personalized spatial spectral abnormalities of high delta and low alpha bands in EEG of patients with early Parkinson's disease. J. Neural Eng. 18:066036. doi: 10.1088/1741-2552/ac40a0, PMID: 34875634

[ref10] Colrain I. M. Nicholas C. L. Baker F. C. (2014). Alcohol and the sleeping brain. Handb. Clin. Neurol. 125, 415–431. doi: 10.1016/B978-0-444-62619-6.00024-0, PMID: 25307588 PMC5821259

[ref11] Conroy D. A. Arnedt J. T. (2014). Sleep and substance use disorders: an update current. Psychiatry Rep. 16, 487–494. doi: 10.1007/s11920-014-0487-3 25135784

[ref12] Coullaut-Valera R. Arbaiza I. Bajo R. Arrúe R. López M. E. Coullaut-Valera J.. (2014). Drug polyconsumption is associated with increased synchronization of brain electrical-activity at rest and in a counting task. Int. J. Neural Syst. 24:1450005. doi: 10.1142/S0129065714500051 24344693

[ref13] Cui Y. Versace F. Engelmann J. M. Minnix J. A. Robinson J. D. Lam C. Y.. (2013). Alpha oscillations in response to affective and cigarette-related stimuli in smokers. Nicotine Tob. Res. 15, 917–924. doi: 10.1093/ntr/nts209, PMID: 23060019 PMC3621581

[ref14] Daubechies I. Lu J. Wu H. T. (2011). Synchrosqueezed wavelet transforms: an empirical mode decomposition-like tool. Appl. Comput. Harmon. Anal. 30, 243–261. doi: 10.1016/j.acha.2010.08.002

[ref15] Delorme A. Makeig S. (2004). EEGLAB: an open source toolbox for analysis of single-trial EEG dynamics including independent component analysis. J. Neurosci. Methods 134, 9–21. doi: 10.1016/j.jneumeth.2003.10.009, PMID: 15102499

[ref16] Ehtasham J. Pierpaolo C. Filippo Z. Cosimo D. G. (2019). Hilbert spectral analysis of EEG data reveals spectral dynamics associated with microstates. J. Neurosci. Methods 325:108317. doi: 10.1016/j.jneumeth.2019.108317 31302155

[ref17] Franken I. Stam C. J. Hendriks V. M. van den Brink W. (2004). Electroencephalographic power and coherence analyses suggest altered brain function in abstient male heroin-dependent patients. Neuropsychobiology 49, 105–110. doi: 10.1159/000076419, PMID: 14981343

[ref18] Fulton T. C. Charlotte A. B. (2009). Impulsivity, frontal lobes and risk for addiction. Pharmacol. Biochem. Behav. 93, 237–247. doi: 10.1016/j.pbb.2009.04.018, PMID: 19410598 PMC2730661

[ref19] Gabeff V. Teijeiro T. Zapater M. Cammoun L. Rheims S. Ryvlin P.. (2021). Interpreting deep learning models for epileptic seizure detection on EEG signals. Artif. Intell. Med. 117:102084. doi: 10.1016/j.artmed.2021.102084, PMID: 34127231

[ref20] Gekht A. B. Polunina A. G. Briun E. A. Davydov D. M. (2002). Brain bioelectrical activities in heroin addicts during early abstinence period. Vserossiiskoe Obshchestvo Psikhiatrov. 442, 86–87. doi: 10.1007/s00428-002-0708-8 12789826

[ref21] Grieder M. Koenig T. Kinoshita T. Utsunomiya K. Wahlund L. O. Dierks T.. (2016). Discovering EEG resting state alterations of semantic dementia. Clin. Neurophysiol. 127, 2175–2181. doi: 10.1016/j.clinph.2016.01.025, PMID: 27072087

[ref22] Hadjileontiadis A. Leontios J. Apostolidis U. Georgios K. (2017). Swarm decomposition: A novel signal analysis using swarm intelligence. Signal Processing: The Official Publication of the EURASIP.

[ref23] Hu S. Lei B. Wang S. Wang Y. Feng Z. Shen Y. (2021). Bidirectional mapping generative adversarial networks for brain MR to PET synthesis. IEEE Trans. Med. Imaging 41, 145–157. doi: 10.1109/TMI.2021.3107013, PMID: 34428138

[ref24] Hu S. Yu W. Chen Z. Wang S. (2020). Medical image reconstruction using generative adversarial network for Alzheimer disease assessment with class-imbalance problem. In: *IEEE International Conference on Computer and Communications*.

[ref25] Huang N. E. (1998). The empirical mode decomposition and the Hilbert spectrum for nonlinear and non-stationary time series analysis. Proc R Soc London 454, 903–995. doi: 10.21105/joss.02977

[ref26] Huang Z. W. Long S. R. Arnold K. C. Chen X. Blank K. Norden E. (2009). On Instantaneous Frequency. Adv. Adapt. Data Anal. 01, 177–229. doi: 10.1142/S1793536909000096

[ref27] John A. R. Cao Z. Chen H. T. Martens K. E. Georgiades M. Gilat M.. (2023). Predicting the onset of freezing of gait using EEG dynamics. Appl. Sci. 13:302. doi: 10.3390/app13010302

[ref28] Kingsley S. Sethukarasi T. (2023). Flower pollination student psychology optimization-integrated context deep learning and probabilistic-based fusion for image inpainting. Int. J. Wavelets Multiresolut Inf. Process. 21:3. doi: 10.1142/S0219691322500503

[ref29] Kiran R. V. Rajagopalan S. S. Bhardwaj S. Panda R. Reddam V. R. Ganne C.. (2018). Machine learning detects EEG microstate alterations in patients living with temporal lobe epilepsy. Seizure 61, 8–13. doi: 10.1016/j.seizure.2018.07.007, PMID: 30044996

[ref30] Koenig T. Lehmann D. Merlo M. C. Kochi K. Hell D. Koukkou M. (1999). A deviant EEG brain microstate in acute, neurolep-tic-naive schizophrenics at rest. Eur. Arch. Psychiatry Clin. Neurosci. 249, 205–211. doi: 10.1007/s004060050088, PMID: 10449596

[ref31] Koenig T. Studer D. Hubl D. Melie L. Strik W. K. (2018). Brain connectivity at different time-scales measured with EEG. Philos. Trans. R. Soc. Lond. B Biol. Sci. 360, 1015–1024. doi: 10.1098/rstb.2005.1649, PMID: 16087445 PMC1854932

[ref32] Lehmann D. (1994). Multichannel topography of human alpha EEG fields. Electroencephalogr. Clin. Neurophysiol. 31, 439–449. doi: 10.1016/0013-4694(71)90165-9, PMID: 4107798

[ref33] Lehmann D. Faber P. L. Galderisi S. (2005). EEG microstate duration and syntax in acute, medication-naïve, first-episode schizo-phrenia: a multi-center study. Psychiatry Res. 138, 141–156. doi: 10.1016/j.pscychresns.2004.05.007 15766637

[ref34] Lehmann D. Ozaki H. Pal I. (1987). EEG alpha map series: rain microstates by space-oriented adaptive segmentation. Electroencephalogr. Clin. Neurophysiol. 67, 271–288. doi: 10.1016/0013-4694(87)90025-3 2441961

[ref35] Linyuan D. Xiaoyi F. (2005). The neurobiological mechanism of nicotine dependence. Adv. Psychol. Sci. 13, 534-543. doi: 10.3969/j.issn.1671-3710.2005.04.018

[ref36] Littel M. Franken I. Strien J. V. (2009). Changes in the electroencephalographic Spectrum in response to smoking cues in smokers and ex-smoker. Neuropsychobiology 59, 43–50. doi: 10.1159/000205517, PMID: 19270463

[ref37] Liu S. Sun Y. He L. Kang Y. (2022). Weak signal processing methods based on improved HHT and filtering techniques for steel wire rope. Appl. Sci. 12:6969. doi: 10.3390/app12146969

[ref38] Michel C. M. Koenig T. (2017). EEG microstates as a tool for studying the temporal dynamics of whole-brain neuronal networks: a review. Neuroimage 180, 577–593. doi: 10.1016/j.neuroimage.2017.11.062, PMID: 29196270

[ref39] Milz P. Pascual-Marqui R. D. Achermann P. Kochi K. Faber P. L. (2017). The EEG microstate topography is predominantly de-termined by intracortical sources in the alpha band. Neuroimage 162, 353–361. doi: 10.1016/j.neuroimage.2017.08.058 28847493

[ref40] Mumtaz W. Vuong P. L. Xia L. Malik A. S. Rashid R. B. A. (2017). An EEG-based machine learning method to screen alcohol use disorder. Cogn. Neurodyn. 11, 161–171. doi: 10.1007/s11571-016-9416-y, PMID: 28348647 PMC5350086

[ref41] Murray M. M. Brunet D. Michel C. M. (2008). Topographic ERP analyses: a step-by-step tutorial review. Brain Topogr. 20, 249–264. doi: 10.1007/s10548-008-0054-5 18347966

[ref42] Olivennes A. Charlesnicolas A. Olievenstein C. (1983). Changes in the waking electroencephalogram in serve heroin addiction. Annales Medico-Psychologiques. 4, 458–469. 6651090

[ref44] Pascual-Marqui R. D. Michel C. M. (1995). Segmentation of brain electrical activity into microstates: model estimation and validation. I.E.E.E. Trans. Biomed. Eng. 42, 658–665. doi: 10.1109/10.391164, PMID: 7622149

[ref45] Pascualmarqui R. D. Michel C. M. Lehmann D. (1994). Low resolution electromagnetic tomography: a new method for localizing electrical activity in the brain international. journal of psychophysiology official. J Int Organ Psychophysiol. 18, 49–65. doi: 10.1016/0167-8760(84)90014-x 7876038

[ref46] Peng R. (2019). Microstate analysis and study of resting state EEG in heroin abusers. Lanzhou: Lanzhou University.

[ref47] Pengfei W. Ruiting Y. Xin M. Hong Z. (2019). Impulse or habit? The nature and mechanism of impulsivity in different stages of addiction. Adv. Psychol. Sci. 5, 834–842. doi: 10.3724/SP.J.1042.2019.00834

[ref48] Poulsen A. T. Pedroni A. Langer N. Hansen L. K. (2020). Microstate EEGlab toolbox:An introductory guide. bioRxiv [Preprint].

[ref49] Prasanth T. Thomas J. Yuvaraj R. Jing J. Cash S. S. Chaudhari R.. (2020). Deep learning for Interictal Epileptiform spike detection from scalp EEG frequency sub bands. Annu Int Conf IEEE Eng Med Biol Soc. 2020, 3703–3706. doi: 10.1109/EMBC44109.2020.9175644, PMID: 33018805 PMC7545315

[ref50] Qu T. Jin J. Xu R. Wang X. Cichocki A. (2022). Riemannian distance based channel selection and feature extraction combining discriminative time-frequency bands and Riemannian tangent space for MI-BCIs. J. Neural Eng. 19:056025. doi: 10.1088/1741-2552/ac9338, PMID: 36126643

[ref51] Reid M. S. Starosta A. Flammino F. Howard B. Prichep L. S. (2004). Quantitative electroencephalographic studies of cue-induced cocaine craving. Clin. EEG Neurosci. 34, 110–123. doi: 10.1177/155005940303400305, PMID: 14521273

[ref52] Robbins T. W. Ersche K. D. Everitt B. J. (2008). Drug addiction and the memory systems of the brain. Ann. N. Y. Acad. Sci. 1141, 1–21. doi: 10.1196/annals.1441.020 18991949

[ref53] Samaha J. Larocque J. J. Postle B. R. (2022). Spontaneous alpha-band amplitude predicts subjective visibility but not discrimination accuracy during high-level perception. Conscious. Cogn. 102:103337. doi: 10.1016/j.concog.2022.103337, PMID: 35525224 PMC9631905

[ref54] Seitzman B. A. Abell M. Bartley S. C. Erickson M. A. Hetrick W. P. (2017). Cognitive manipulation of brain electric microstates. Neuroimage 146, 533–543. doi: 10.1016/j.neuroimage.2016.10.002, PMID: 27742598 PMC5321823

[ref55] Shinan S. (2020). Neural basis of smoking cue response and prediction of therapeutic effect of neurofeedback intervention. Tianjin: Tianjin Normal University.

[ref56] Shuaiyang L. (2021). Study on intracranial pressure detection technique based on feature fusion of resting state EEG signals. Zhengzhou: Zhengzhou University.

[ref57] Strik W. K. Chiaramonti R. Muscas G. C. Paganini M. Mueller T. J. Fallgatter A. J.. (1997). Decreased EEG microstate duration and anteriorisation of the brain electrical fields in mild and moderate dementia of the Alzheimer type psychiatry research. Neuroimaging 75, 183–191. doi: 10.1016/s0925-4927(97)00054-1, PMID: 9437775

[ref58] Světlák M. Bob P. Erník M. Chládek J. Kukleta M. (2010). Electrodermal dimensional complexity and smoking. *Scripta Medica. Brno: Masarykova univerzita, roč*. 83, 63–69, 6.

[ref59] Thakur G. Brevdo E. Fučkar N. S. Wu H. T. (2013). The Synchrosqueezing algorithm for time-varying spectral analysis: robustness properties and new paleoclimate applications. Signal Process. 93, 1079–1094. doi: 10.1016/j.sigpro.2012.11.029

[ref60] Thomas K. Leslie P. Lehmann D. Pedro V. S. Elisabeth B. Horst K.. (2002). Millisecond by millisecond, year by year: normative EEG microstates and developmental stages. Neuroimage 16, 41–48. doi: 10.1006/nimg.2002.1070, PMID: 11969316

[ref61] Totev T. Taskov T. Dushanova J. (2023). A wireless EEG system for Neurofeedback training. Appl. Sci. 13:96. doi: 10.3390/app13010096

[ref62] Uyulan C. Ergüzel T. T. Unubol H. Cebi M. Sayar G. H. Nezhad Asad M.. (2020). Major depressive disorder classification based on different convolutional neural network models: deep learning approach. Clin. EEG Neurosci. 52, 38–51. doi: 10.1177/1550059420916634, PMID: 32491928

[ref63] Wacker M. Witte A. (2011). The matched Gabor transform—a tool for adaptive phase extraction. Front. Comput. Neurosci. 5:00223. doi: 10.3389/conf.fncom.2011.53.00223

[ref64] Walther T. G. (2005). Cluster validation by prediction strength. J. Comput. Graph. Stat. 3, 511–528. doi: 10.1198/106186005X59243

[ref65] Wei T. Taolin C. Xiaoqi H. (2017). Background versus event craving: differentiating the different pathways of psychological craving for nicotine addiction. Adv. Psychol. Sci. 11, 1932–1941. doi: 10.3724/SP.J.1042.2017.01932

[ref66] Weifeng L. Xiaoming L. Ruomeng D. Xiaoying T. (2017). Exploring differences between left and right hand motor imagery via spatio-temporal EEG microstate. Computer Assist Surg 22, 258–266. doi: 10.1080/24699322.2017.1389404, PMID: 29096552

[ref43] World Health Organization (2009). International statistical classification of diseases and related health problems (the) ICD-10. Acta Chir. Iugosl. 56, 65–69. doi: 10.2298/ACI0903065V 20218105

[ref67] Yan Y. Dahmani L. Ren J. Shen L. Peng X. Wang R.. (2020). Reconstructing lost BOLD signal in individual participants using deep machine learning. Nat. Commun. 11:5046. doi: 10.1038/s41467-020-18823-9, PMID: 33028816 PMC7542429

[ref68] Yan Xue X. Jia Hui D. YaYun C. LiBo Z. Ping W. (2017). Effect of selective inhibition of reactivated nicotine-associated memories with propranolol on nicotine craving. JAMA Psychiat. 74, 224–232. doi: 10.1001/jamapsychiatry.2016.3907, PMID: 28146250 PMC6201291

[ref69] Yang W. C. Zhang P. L. Wu D. H. Xin Z. (2013). A method of false component discriminant of EMD based on Kolmogo-rov-Smirnov test. Appl. Mech. Mater. 427-429, 2005–2008. doi: 10.4028/www.scientific.net/AMM.427-429.2005

[ref70] You S. Lei B. Wang S. Chui C. K. Cheung A. C. Liu Y.. (2022). Fine perceptive GANs for brain MR image super-resolution in wavelet domain. IEEE Trans Neural Netw Learn Syst. 4, 1–13. doi: 10.1109/TNNLS.2022.3153088 35254996

[ref71] Zernig G. Ahmed S. H. Cardinal R. N. Morgan D. Acquas E. Foltin R. W.. (2007). Explaining the escalation of drug use in substance dependence: models and appropriate animal laboratory tests. Pharmacology 80, 65–119. doi: 10.1159/000103923, PMID: 17570954

