# Supplementary material for: Improved HHT-microstate analysis of EEG in nicotine addicts
Source: Front Neurosci. 2023 May 24;17:1174399. doi: 10.3389/fnins.2023.1174399 (PMC10244792; doi:10.3389/fnins.2023.1174399)
Supplement: Supplementary file 1 [file Data_Sheet_1.docx]

Supplementary Material

Improved HHT-Microstate Analysis of EEG in Nicotine Addicts

Xin Xiong, Jiannan Feng, Yaru Zhang, Di Wu, Sanli Yi, Chunwu Wang, Ruixiang Liu, Jianfeng He*

*** Correspondence:** Jianfeng He: jfenghe@foxmail.com

# Supplementary Tables

## Supplementary Tables A

Supplementary Tables A shows the results of one-way ANOVA for each microstate parameter between neutral and smoke group at delta, alpha and beta bands under the improved HHT-Microstate method.

**Supplementary Table 1.** MD of each microstate at each frequency band.

| **Subjects** | **Microstates** | **neutral** | **smoke** | **F value** | **η^2^** | **p value** |
| --- | --- | --- | --- | --- | --- | --- |
| Delta [0.1~4Hz] | D1 | 127.66±10.97 | 131.01±14.50 | 0.678 | 112.04 | 0.17634 |
|  | D2 | 125.50±7.63 | 127.18±7.83 | 0.470 | 28.29 | 0.27774 |
|  | D3 | 123.63±9.19 | 126.21±6.37 | 1.065 | 66.54 | 0.20296 |
|  | D4 | 127.82±23.78 | 125.16±7.34 | 0.228 | 70.55 | 0.32972 |
|  | D5 | 130.38±34.43 | 121.22±5.64 | 1.381 | 840.19 | 0.12762 |
| Alpha [8~12Hz] | A1 | 108.60±6.93 | 105.67±5.46 | 2.209 | 85.97 | 0.07383 |
|  | A2 | 101.13±6.60 | 104.07±3.83 | 2.996 | 86.32 | 0.06739 |
|  | A3 | 100.27±6.50 | 102.84±5.59 | 1.800 | 66.15 | 0.09931 |
|  | A4 | 97.52±4.45 | 97.24±6.64 | 0.026 | 0.814 | 0.43348 |
|  | A5 | 96.73±5.82 | 95.94±4.41 | 0.232 | 6.20 | 0.32464 |
| Beta [12~30Hz] | B1 | 87.81±6.92 | 83.90±16.97 | 0.911 | 153.10 | 0.19098 |
|  | B2 | 80.62±5.13 | 80.14±14.76 | 0.019 | 2.28 | 0.44395 |
|  | B3 | 80.50±3.65 | 76.48±14.88 | 1.378 | 161.60 | 0.13253 |
|  | B4 | 76.90±5.42 | 76.24±15.44 | 0.032 | 4.26 | 0.42295 |

**Supplementary Table 2.** OPS of each microstate at each frequency band.

| **Subjects** | **Microstates** | **neutral** | **smoke** | **F value** | **η^2^** | **p value** |
| --- | --- | --- | --- | --- | --- | --- |
| Delta [0.1~4Hz] | D1 | 1.62±0.39 | 1.63±0.40 | 0.011 | 0.002 | 0.46129 |
|  | D2 | 1.49±0.49 | 1.68±0.20 | 2.574 | 0.363 | 0.05431 |
|  | D3 | 1.45±0.42 | 1.59±0.16 | 1.954 | 0.196 | 0.09519 |
|  | D4 | 1.57±0.28 | 1.53±0.20 | 0.259 | 0.015 | 0.29133 |
|  | D5 | 1.59±0.30 | 1.44±0.20 | 3.613 | 0.231 | 0.00436* |
| Alpha [8~12Hz] | A1 | 2.20±0.20 | 2.21±0.17 | 0.013 | 0.001 | 0.44551 |
|  | A2 | 2.03±0.22 | 2.14±0.15 | 3.626 | 0.126 | 0.04725* |
|  | A3 | 1.96±0.19 | 2.01±0.17 | 0.603 | 0.020 | 0.17077 |
|  | A4 | 1.85±0.18 | 1.79±0.15 | 1.684 | 0.048 | 0.07235 |
|  | A5 | 1.81±0.22 | 1.70±0.15 | 3.728 | 0.129 | 0.02972* |
| Beta [12~30Hz] | B1 | 3.39±0.16 | 3.88±2.70 | 0.674 | 2.46 | 0.21021 |
|  | B2 | 3.11±0.45 | 3.81±2.58 | 1.411 | 4.85 | 0.12413 |
|  | B3 | 3.08±0.29 | 3.37±1.96 | 0.423 | 0.83 | 0.26763 |
|  | B4 | 2.65±0.34 | 3.13±2.13 | 0.971 | 2.27 | 0.17580 |

**Supplementary Table 3.** TCR of each microstate at each frequency band.

| **Subjects** | **Microstates** | **neutral** | **smoke** | **F value** | **η^2^** | **p value** |
| --- | --- | --- | --- | --- | --- | --- |
| Delta [0.1~4Hz] | D1 | 0.21±0.06 | 0.22±0.07 | 0.203 | 0.001 | 0.33170 |
|  | D2 | 0.19±0.07 | 0.21±0.03 | 2.276 | 0.006 | 0.08287 |
|  | D3 | 0.18±0.06 | 0.20±0.03 | 2.127 | 0.004 | 0.10447 |
|  | D4 | 0.21±0.09 | 0.19±0.03 | 0.440 | 0.002 | 0.26045 |
|  | D5 | 0.22±0.11 | 0.18±0.03 | 2.603 | 0.016 | 0.03856* |
| Alpha [8~12Hz] | A1 | 0.24±0.03 | 0.23±0.02 | 0.504 | 0.001 | 0.22850 |
|  | A2 | 0.21±0.03 | 0.22±0.02 | 4.214 | 0.003 | 0.03968* |
|  | A3 | 0.20±0.03 | 0.21±0.02 | 1.341 | 0.001 | 0.08526 |
|  | A4 | 0.18±0.02 | 0.17±0.22 | 1.032 | 0.001 | 0.10422 |
|  | A5 | 0.18±0.03 | 0.16±0.02 | 2.616 | 0.002 | 0.06824 |
| Beta [12~30Hz] | B1 | 0.30±0.03 | 0.29±0.03 | 1.022 | 0.001 | 0.08877 |
|  | B2 | 0.25±0.04 | 0.27±0.02 | 3.517 | 0.004 | 0.02327* |
|  | B3 | 0.25±0.03 | 0.23±0.03 | 2.669 | 0.002 | 0.06070 |
|  | B4 | 0.20±0.03 | 0.21±0.03 | 0.639 | 0.001 | 0.24544 |

**Supplementary Table 4.** GEV of each microstate at each frequency band.

| **Subjects** | **Microstates** | **neutral** | **smoke** | **F value** | **η^2^** | **p value** |
| --- | --- | --- | --- | --- | --- | --- |
| Delta [0.1~4Hz] | D1 | 19.30±6.60 | 20.33±9.49 | 0.157 | 0.001 | 0.30099 |
|  | D2 | 11.77±2.33 | 12.54±3.79 | 0.606 | 0.001 | 0.21654 |
|  | D3 | 9.55±1.90 | 9.26±1.93 | 0.224 | 0.001 | 0.28415 |
|  | D4 | 9.19±3.66 | 8.27±1.96 | 0.975 | 0.001 | 0.14192 |
|  | D5 | 7.75±2.49 | 6.64±1.81 | 2.604 | 0.001 | 0.00509* |
| Alpha [8~12Hz] | A1 | 9.64±2.22 | 9.56±1.50 | 0.018 | 0.001 | 0.42914 |
|  | A2 | 7.62±1.65 | 8.62±1.65 | 3.673 | 0.001 | 0.03696* |
|  | A3 | 7.18±1.90 | 7.41±1.47 | 0.187 | 0.001 | 0.25183 |
|  | A4 | 6.13±0.82 | 5.97±1.03 | 0.317 | 0.001 | 0.23322 |
|  | A5 | 5.69±1.69 | 5.16±0.90 | 1.503 | 0.001 | 0.11123 |
| Beta [12~30Hz] | B1 | 9.68±3.47 | 9.48±3.55 | 0.033 | 0.001 | 0.27022 |
|  | B2 | 7.47±1.89 | 8.67±3.53 | 1.778 | 0.001 | 0.02874* |
|  | B3 | 6.60±1.17 | 6.35±1.39 | 0.371 | 0.001 | 0.20029 |
|  | B4 | 4.91±1.18 | 5.25±1.33 | 0.698 | 0.001 | 0.16015 |

## Supplementary Tables B

Supplementary Tables B show the microstate parameters and Riemann distance at each frequency band under FIR method.

**Supplementary Table 1.** Microstate parameters at each frequency band under FIR-Microstate method.

| **Subjects** | **Microstates** | **MD** | | **OPS** | | **TCR** | | **GEV** | |
| --- | --- | --- | --- | --- | --- | --- | --- | --- | --- |
|  |  | neutral | smoke | neutral | smoke | neutral | smoke | neutral | smoke |
| Delta | D1 | 93.23±25.21 | 94.34±35.01 | 2.61±1.01 | 1.89±1.03 | 0.24±0.10 | 0.18±0.11 | 10.16±6.11 | 9.17±8.28 |
|  | D2 | 79.23±26.29 | 102.46±39.99 | 2.24±0.90 | 2.08±0.85 | 0.19±0.09 | 0.21±0.09 | 7.83±6.93 | 9.08±7.45 |
|  | D3 | 96.02±29.12 | 100.66±47.05 | 2.16±0.87 | 2.19±1.11 | 0.21±0.12 | 0.22±0.13 | 6.04±6.53 | 6.60±4.35 |
|  | D4 | 93.66±38.21 | 89.61±59.04 | 2.00±0.86 | 1.77±0.90 | 0.19±0.12 | 0.18±0.14 | 3.83±2.10 | 3.54±3.00 |
|  | D5 | 93.42±62.21 | 103.91±56.26 | 1.84±0.72 | 1.97±0.87 | 0.16±0.10 | 0.21±0.16 | 2.57±1.70 | 3.81±2.68 |
| Theta | T1 | 77.83±7.76 | 78.62±8.33 | 3.28±0.35 | 3.36±0.35 | 0.26±0.04 | 0.27±0.05 | 9.58±2.23 | 9.83±2.36 |
|  | T2 | 73.93±6.99 | 72.89±6.36 | 2.96±0.42 | 2.92±0.40 | 0.22±0.03 | 0.21±0.04 | 7.63±1.66 | 7.33±1.61 |
|  | T3 | 73.01±6.14 | 70.51±4.37 | 2.83±0.36 | 2.77±0.54 | 0.21±0.04 | 0.20±0.04 | 6.85±1.42 | 6.59±1.65 |
|  | T4 | 70.04±7.38 | 68.49±6.71 | 2.51±0.37 | 2.41±0.32 | 0.18±0.03 | 0.17±0.03 | 5.53±1.14 | 5.21±1.12 |
|  | T5 | 65.66±6.86 | 66.45±6.03 | 2.19±0.43 | 2.38±0.41 | 0.14±0.03 | 0.16±0.04 | 4.12±1.02 | 4.50±1.10 |
| Alpha | A1 | 77.85±6.54 | 80.07±6.39 | 3.12±0.36 | 3.11±0.46 | 0.24±0.03 | 0.25±0.04 | 9.13±1.47 | 9.57±2.12 |
|  | A2 | 73.94±5.39 | 74.75±6.75 | 2.92±0.33 | 2.99±0.30 | 0.22±0.03 | 0.22±0.03 | 7.92±1.57 | 7.92±1.47 |
|  | A3 | 71.05±3.96 | 71.40±5.54 | 2.78±0.28 | 2.66±0.39 | 0.20±0.02 | 0.19±0.03 | 6.57±0.95 | 6.42±1.40 |
|  | A4 | 73.25±5.95 | 68.06±4.87 | 2.57±0.33 | 2.55±0.39 | 0.19±0.03 | 0.17±0.03 | 6.11±1.36 | 5.66±1.27 |
|  | A5 | 67.52±4.89 | 69.56±6.31 | 2.29±0.34 | 2.34±0.36 | 0.15±0.03 | 0.16±0.03 | 4.61±4.23 | 4.91±1.20 |
| Beta | B1 | 93.06±24.31 | 89.72±14.20 | 3.24±0.68 | 3.45±0.48 | 0.29±0.05 | 0.31±0.04 | 9.14±4.30 | 9.94±5.86 |
|  | B2 | 89.79±28.19 | 89.37±36.46 | 3.18±0.63 | 3.25±0.59 | 0.27±0.04 | 0.27±0.05 | 7.55±2.01 | 7.62±1.83 |
|  | B3 | 78.58±6.22 | 76.79±6.12 | 3.01±0.36 | 3.10±0.34 | 0.24±0.03 | 0.24±0.05 | 5.81±1.10 | 5.91±1.33 |
|  | B4 | 75.43±8.77 | 71.26±10.31 | 2.69±0.36 | 2.56±0.48 | 0.20±0.03 | 0.18±0.04 | 4.45±1.19 | 4.03±1.09 |
| Gamma | G1 | 119.50±61.73 | 145.15±123.87 | 2.86±0.97 | 2.65±1.01 | 0.31±0.10 | 0.32±0.10 | 11.62±5.93 | 12.20±6.53 |
|  | G2 | 121.96±155.53 | 106.96±107.01 | 2.69±0.74 | 2.77±0.84 | 0.26±0.11 | 0.26±0.12 | 7.40±3.72 | 6.75±2.62 |
|  | G3 | 87.80±20.34 | 82.70±13.63 | 2.73±0.54 | 2.85±0.56 | 0.24±0.08 | 0.24±0.06 | 5.30±2.87 | 4.85±1.20 |
|  | G4 | 102.31±106.54 | 77.82±12.59 | 2.32±0.82 | 2.40±0.57 | 0.19±0.06 | 0.19±0.06 | 3.50±1.17 | 3.53±1.21 |

**Supplementary Table 2.** Riemann distance parameters at each frequency band under HHT-Riemann method.

| **Bands** | **dis_AIRM** | | **dis_Stein** | | **dis_Jeffrey** | | **dis_LogED** | |
| --- | --- | --- | --- | --- | --- | --- | --- | --- |
|  | neutral | smoke | neutral | smoke | neutral | smoke | neutral | smoke |
| Delta | 7.63±2.13 | 7.59±1.60 | 2.40±0.54 | 2.39±0.41 | 8.67±7.33 | 8.38±5.47 | 6.98±2.06 | 6.95±1.57 |
| Theta | 6.09±1.05 | 6.11±1.12 | 1.98±0.30 | 1.99±0.32 | 5.54±1.64 | 5.59±1.84 | 5.52±1.01 | 5.52±1.09 |
| Alpha | 6.01±1.06 | 6.02±1.08 | 1.96±0.30 | 1.96±0.31 | 5.36±1.57 | 5.44±1.61 | 5.47±1.02 | 5.46±1.04 |
| Beta | 6.04±1.42 | 5.83±1.31 | 1.96±0.40 | 1.90±0.36 | 5.50±2.05 | 5.29±2.07 | 5.70±1.243 | 5.49±1.33 |
| Gamma | 6.63±1.85 | 6.32±1.66 | 2.12±0.51 | 2.03±0.45 | 6.51±3.06 | 6.08±2.90 | 6.36±1.84 | 6.04±1.66 |

**Supplementary Table 3.** Riemann distance parameters at each frequency band under FIR-Riemann method.

| **Bands** | **dis_AIRM** | | **dis_Stein** | | **dis_Jeffrey** | | **dis_LogED** | |
| --- | --- | --- | --- | --- | --- | --- | --- | --- |
|  | neutral | smoke | neutral | smoke | neutral | smoke | neutral | smoke |
| Delta | 12.42±1.96 | 12.36±1.88 | 3.60±0.41 | 3.58±0.39 | 23.51±19.22 | 22.76±19.45 | 11.22±1.75 | 11.14±1.71 |
| Theta | 7.65±1.31 | 7.68±1.31 | 2.37±0.32 | 2.38±0.32 | 9.15±5.31 | 9.18±5.35 | 6.59±1.15 | 6.60±1.16 |
| Alpha | 7.92±1.35 | 7.90±1.33 | 2.46±0.34 | 2.45±0.33 | 9.17±5.00 | 9.26±5.18 | 6.99±1.16 | 6.94±1.13 |
| Beta | 9.28±2.30 | 8.66±1.89 | 2.82±0.58 | 2.66±0.47 | 11.76±6.09 | 10.68±6.41 | 8.70±2.28 | 7.98±1.82 |
| Gamma | 10.56±3.14 | 10.04±2.82 | 3.12±0.78 | 3.00±0.70 | 15.48±9.04 | 14.14±8.88 | 10.13±3.09 | 9.64±2.80 |

## Supplementary Tables C

Supplementary Tables C show the one-way ANOVA results at each frequency band under FIR-Microstate and FIR-Riemann method where supplementary table 5 to supplementary table 8 are the results under HHT-Riemann, and supplementary table 9 to supplementary table 12 are the results under FIR-Riemann.

**Supplementary Table 1.** MD of each microstate at each frequency band.

| **Subjects** | **Microstates** | **neutral** | **smoke** | **F value** | **η^2^** | **p value** |
| --- | --- | --- | --- | --- | --- | --- |
| Delta [0.1~4Hz] | D1 | 93.23±25.21 | 94.34±35.01 | 0.013 | 12.21 | 0.45929 |
|  | D2 | 79.23±26.29 | 102.46±39.99 | 4.711 | 5396.26 | 0.01530* |
|  | D3 | 96.02±29.12 | 100.66±47.05 | 0.141 | 215.33 | 0.31820 |
|  | D4 | 93.66±38.21 | 89.61±59.04 | 0.066 | 163.92 | 0.39899 |
|  | D5 | 93.42±62.21 | 103.91±56.26 | 0.313 | 1100.48 | 0.30068 |
| Alpha [8~12Hz] | A1 | 77.85±6.54 | 80.07±6.39 | 1.185 | 49.50 | 0.11335 |
|  | A2 | 73.94±5.39 | 74.75±6.75 | 0.173 | 6.45 | 0.28678 |
|  | A3 | 71.05±3.96 | 71.40±5.54 | 0.053 | 1.23 | 0.40718 |
|  | A4 | 73.25±5.95 | 68.06±4.87 | 9.097 | 268.75 | 0.00258* |
|  | A5 | 67.52±4.89 | 69.56±6.31 | 1.308 | 41.62 | 0.13577 |
| Beta [12~30Hz] | B1 | 93.06±24.31 | 89.72±14.20 | 0.054 | 59.84 | 0.29893 |
|  | B2 | 89.79±28.19 | 89.37±36.46 | 0.377 | 14.34 | 0.47132 |
|  | B3 | 78.58±6.22 | 76.79±6.12 | 1.003 | 31.54 | 0.17716 |
|  | B4 | 75.43±8.77 | 71.26±10.31 | 0.184 | 6.71 | 0.01469* |

**Supplementary Table 2.** OPS of each microstate at each frequency band.

| **Subjects** | **Microstates** | **neutral** | **smoke** | **F value** | **η^2^** | **p value** |
| --- | --- | --- | --- | --- | --- | --- |
| Delta [0.1~4Hz] | D1 | 2.61±1.01 | 1.89±1.03 | 5.087 | 5.31 | 0.00893* |
|  | D2 | 2.24±0.90 | 2.08±0.85 | 0.356 | 0.27 | 0.25153 |
|  | D3 | 2.16±0.87 | 2.19±1.11 | 0.008 | 0.008 | 0.45705 |
|  | D4 | 2.00±0.86 | 1.77±0.90 | 0.694 | 0.54 | 0.17897 |
|  | D5 | 1.84±0.72 | 1.97±0.87 | 0.250 | 0.16 | 0.33820 |
| Alpha [8~12Hz] | A1 | 3.12±0.36 | 3.11±0.46 | 0.006 | 0.001 | 0.46335 |
|  | A2 | 2.92±0.33 | 2.99±0.30 | 0.407 | 0.04 | 0.17967 |
|  | A3 | 2.78±0.28 | 2.66±0.39 | 1.251 | 0.14 | 0.07707 |
|  | A4 | 2.57±0.33 | 2.55±0.39 | 0.023 | 0.003 | 0.44113 |
|  | A5 | 2.29±0.34 | 2.34±0.36 | 0.200 | 0.03 | 0.33031 |
| Beta [12~30Hz] | B1 | 3.24±0.68 | 3.45±0.48 | 0.005 | 0.002 | 0.15171 |
|  | B2 | 3.18±0.63 | 3.25±0.59 | 0.280 | 0.04 | 0.31095 |
|  | B3 | 3.01±0.36 | 3.10±0.34 | 0.294 | 0.04 | 0.21135 |
|  | B4 | 2.69±0.36 | 2.56±0.48 | 1.270 | 0.18 | 0.15952 |

**Supplementary Table 3.** TCR of each microstate at each frequency band.

| **Subjects** | **Microstates** | **neutral** | **smoke** | **F value** | **η^2^** | **p value** |
| --- | --- | --- | --- | --- | --- | --- |
| Delta [0.1~4Hz] | D1 | 0.24±0.10 | 0.18±0.11 | 3.327 | 0.038 | 0.03406* |
|  | D2 | 0.19±0.09 | 0.21±0.09 | 0.413 | 0.003 | 0.22450 |
|  | D3 | 0.21±0.12 | 0.22±0.13 | 0.111 | 0.002 | 0.34923 |
|  | D4 | 0.19±0.12 | 0.18±0.14 | 0.122 | 0.002 | 0.36542 |
|  | D5 | 0.16±0.10 | 0.21±0.16 | 1.096 | 0.02 | 0.19016 |
| Alpha [8~12Hz] | A1 | 0.24±0.03 | 0.25±0.04 | 0.285 | 0.001 | 0.27205 |
|  | A2 | 0.22±0.03 | 0.22±0.03 | 0.508 | 0.001 | 0.14278 |
|  | A3 | 0.20±0.02 | 0.19±0.03 | 0.590 | 0.001 | 0.18853 |
|  | A4 | 0.19±0.03 | 0.17±0.03 | 2.080 | 0.002 | 0.05964 |
|  | A5 | 0.15±0.03 | 0.16±0.03 | 0.746 | 0.001 | 0.19058 |
| Beta [12~30Hz] | B1 | 0.29±0.05 | 0.31±0.04 | 0.097 | 0.001 | 0.09634 |
|  | B2 | 0.27±0.04 | 0.27±0.05 | 0.460 | 0.001 | 0.45747 |
|  | B3 | 0.24±0.03 | 0.24±0.05 | 0.849 | 0.001 | 0.42331 |
|  | B4 | 0.20±0.03 | 0.18±0.04 | 0.680 | 0.001 | 0.04239* |

**Supplementary Table 4.** GEV of each microstate at each frequency band.

| **Subjects** | **Microstates** | **neutral** | **smoke** | **F value** | **η^2^** | **p value** |
| --- | --- | --- | --- | --- | --- | --- |
| Delta [0.1~4Hz] | D1 | 10.16±6.11 | 9.17±8.28 | 0.185 | 0.001 | 0.33034 |
|  | D2 | 7.83±6.93 | 9.08±7.45 | 0.302 | 0.002 | 0.18847 |
|  | D3 | 6.04±6.53 | 6.60±4.35 | 0.102 | 0.001 | 0.36582 |
|  | D4 | 3.83±2.10 | 3.54±3.00 | 0.124 | 0.001 | 0.35943 |
|  | D5 | 2.57±1.70 | 3.81±2.68 | 3.046 | 0.002 | 0.06707 |
| Alpha [8~12Hz] | A1 | 9.13±1.47 | 9.57±2.12 | 0.582 | 0.001 | 0.16804 |
|  | A2 | 7.92±1.57 | 7.92±1.47 | 0.001 | 0.001 | 0.49745 |
|  | A3 | 6.57±0.95 | 6.42±1.40 | 0.155 | 0.001 | 0.32873 |
|  | A4 | 6.11±1.36 | 5.66±1.27 | 1.167 | 0.001 | 0.08996 |
|  | A5 | 4.61±4.23 | 4.91±1.20 | 0.612 | 0.001 | 0.18240 |
| Beta [12~30Hz] | B1 | 9.14±4.30 | 9.94±5.86 | 0.029 | 0.001 | 0.08857 |
|  | B2 | 7.55±2.01 | 7.62±1.83 | 0.204 | 0.001 | 0.44519 |
|  | B3 | 5.81±1.10 | 5.91±1.33 | 0.144 | 0.001 | 0.13253 |
|  | B4 | 4.45±1.19 | 4.03±1.09 | 0.052 | 0.001 | 0.42295 |

**Supplementary Table 5.** dis_AIRM of each Riemann at each frequency band.

| **Bands** | **neutral** | **smoke** | **F value** | **η^2^** | **p value** |
| --- | --- | --- | --- | --- | --- |
| Delta | 12.42±1.96 | 12.36±1.88 | 0.042 | 3.69 | 0.83801 |
| Theta | 7.65±1.31 | 7.68±1.31 | 0.016 | 1.721 | 0.89802 |
| Alpha | 7.92±1.35 | 7.90±1.33 | 0.016 | 1.803 | 0.90121 |
| Beta | 9.28±2.30 | 8.66±1.89 | 5.219 | 4.430 | 0.02311* |
| Gamma | 10.56±3.14 | 10.04±2.82 | 1.761 | 8.915 | 0.18622 |

**Supplementary Table 6.** dis_Stein of each Riemann at each frequency band.

| **Bands** | **neutral** | **smoke** | **F value** | **η^2^** | **p value** |
| --- | --- | --- | --- | --- | --- |
| Delta | 3.60±0.41 | 3.58±0.39 | 0.047 | 0.161 | 0.82801 |
| Theta | 2.37±0.32 | 2.38±0.32 | 0.021 | 0.104 | 0.88603 |
| Alpha | 2.46±0.34 | 2.45±0.33 | 0.049 | 0.110 | 0.82515 |
| Beta | 2.82±0.58 | 2.66±0.47 | 5.431 | 0.280 | 0.02103* |
| Gamma | 3.12±0.78 | 3.00±0.70 | 1.523 | 0.549 | 0.21871 |

**Supplementary Table 7.** dis_Jeffrey of each Riemann at each frequency band.

| **Bands** | **neutral** | **smoke** | **F value** | **η^2^** | **p value** |
| --- | --- | --- | --- | --- | --- |
| Delta | 23.51±19.22 | 22.76±19.45 | 0.089 | 373.908 | 0.76501 |
| Theta | 9.15±5.31 | 9.18±5.35 | 0.003 | 28.422 | 0.95944 |
| Alpha | 9.17±5.00 | 9.26±5.18 | 0.021 | 25.940 | 0.88634 |
| Beta | 11.76±6.09 | 10.68±6.41 | 1.775 | 39.101 | 0.18465 |
| Gamma | 15.48±9.04 | 14.14±8.88 | 1.347 | 80.349 | 0.24707 |

**Supplementary Table 8.** dis_LogED of each Riemann at each frequency band.

| **Bands** | **neutral** | **smoke** | **F value** | **η^2^** | **p value** |
| --- | --- | --- | --- | --- | --- |
| Delta | 11.22±1.75 | 11.14±1.71 | 0.142 | 2.981 | 0.70601 |
| Theta | 6.59±1.15 | 6.60±1.16 | 0.004 | 1.334 | 0.95208 |
| Alpha | 6.99±1.16 | 6.94±1.13 | 0.130 | 1.305 | 0.71923 |
| Beta | 8.70±2.28 | 7.98±1.82 | 7.323 | 4.256 | 0.00782* |
| Gamma | 10.13±3.09 | 9.64±2.80 | 1.638 | 8.692 | 0.20231 |

**Supplementary Table 9.** dis_AIRM of each Riemann at each frequency band.

| **Bands** | **neutral** | **smoke** | **F value** | **η^2^** | **p value** |
| --- | --- | --- | --- | --- | --- |
| Delta | 7.63±2.13 | 7.59±1.60 | 0.022 | 3.549 | 0.88104 |
| Theta | 6.09±1.05 | 6.11±1.12 | 0.019 | 1.182 | 0.89102 |
| Alpha | 6.01±1.06 | 6.02±1.08 | 0.015 | 1.152 | 0.90211 |
| Beta | 6.04±1.42 | 5.83±1.31 | 1.440 | 1.577 | 0.21008 |
| Gamma | 6.63±1.85 | 6.32±1.66 | 1.901 | 3.077 | 0.16982 |

**Supplementary Table 10.** dis_Stein of each Riemann at each frequency band.

| **Bands** | **neutral** | **smoke** | **F value** | **η^2^** | **p value** |
| --- | --- | --- | --- | --- | --- |
| Delta | 2.40±0.54 | 2.39±0.41 | 0.012 | 0.227 | 0.91401 |
| Theta | 1.98±0.30 | 1.99±0.32 | 0.007 | 0.093 | 0.93503 |
| Alpha | 1.96±0.30 | 1.96±0.31 | 0.001 | 0.093 | 0.98206 |
| Beta | 1.96±0.40 | 1.90±0.36 | 2.043 | 0.114 | 0.15461 |
| Gamma | 2.12±0.51 | 2.03±0.45 | 1.938 | 0.230 | 0.16554 |

**Supplementary Table 11.** dis_Jeffrey of each Riemann at each frequency band.

| **Bands** | **neutral** | **smoke** | **F value** | **η^2^** | **p value** |
| --- | --- | --- | --- | --- | --- |
| Delta | 8.67±7.33 | 8.38±5.47 | 0.125 | 41.810 | 0.72401 |
| Theta | 5.54±1.64 | 5.59±1.84 | 0.067 | 3.033 | 0.79527 |
| Alpha | 5.36±1.57 | 5.44±1.61 | 0.124 | 2.522 | 0.72533 |
| Beta | 5.50±2.05 | 5.29±2.07 | 0.376 | 3.429 | 0.54091 |
| Gamma | 6.51±3.06 | 6.08±2.90 | 1.222 | 8.883 | 0.27037 |

**Supplementary Table 12.** dis_LogED of each Riemann at each frequency band.

| **Bands** | **neutral** | **smoke** | **F value** | **η^2^** | **p value** |
| --- | --- | --- | --- | --- | --- |
| Delta | 6.98±2.06 | 6.95±1.57 | 0.015 | 3.360 | 0.90201 |
| Theta | 5.52±1.01 | 5.52±1.09 | 0.005 | 1.104 | 0.94322 |
| Alpha | 5.47±1.02 | 5.46±1.04 | 0.001 | 1.068 | 0.97802 |
| Beta | 5.70±1.243 | 5.49±1.33 | 0.027 | 1.425 | 0.86928 |
| Gamma | 6.36±1.84 | 6.04±1.66 | 1.918 | 3.081 | 0.16753 |
